# Norms, prices, and commitment: A comprehensive overview of field experiments in the energy domain and treatment effect moderators

**DOI:** 10.3389/fpsyg.2022.967318

**Published:** 2022-11-08

**Authors:** Stepan Vesely, Christian A. Klöckner, Giuseppe Carrus, Lorenza Tiberio, Federica Caffaro, Mehmet Efe Biresselioglu, Andrea C. Kollmann, Anca C. Sinea

**Affiliations:** ^1^Department of Psychology, Norwegian University of Science of Technology, Trondheim, Norway; ^2^Department of Education, University Roma Tre, Rome, Italy; ^3^Sustainable Energy Division, Izmir University of Economics, Izmir, Turkey; ^4^Energieinstitut an der Johannes Kepler Universität Linz, Linz, Austria; ^5^Department of Political, Administrative and Communication Studies, Babeş-Bolyai University, Cluj-Napoca, Romania

**Keywords:** energy conservation, energy efficiency, social norms, incentives, commitment, goal setting, interventions, moderators

## Abstract

This paper provides a comprehensive overview of field experiments utilizing social norms, commitment and price-based interventions to promote energy conservation, load shifting, and energy efficiency behaviors. Treatment effects reported in the extant literature, as well as the factors that may strengthen or dampen these effects are reviewed. We find that social norm and incentive-based interventions mostly achieve small reductions in energy consumption, and that the effects of commitment-based interventions are essentially zero for the most part. Incentive effects on energy efficiency investments are mostly non-existent, safe for a few exceptions. One gap that we identify is the almost complete absence of field experiments leveraging social norms or commitment to promote energy efficiency investments. We discuss a broad range of (mostly under-researched) plausible moderators of the interventions' effects. Crucially, a more careful attention to moderators in future research can highlight instances in which interventions can be effective, notwithstanding their modest or non-existent average treatment effects. Our review offers a starting point in this regard.

## Introduction

It has long been theorized that social norms, prices and commitment are important determinants of people's decisions, including in the energy domain. More recently, policy makers and other practitioners are becoming increasingly interested in the possibility of harnessing these factors to promote desirable behaviors on a large scale. These behaviors include energy conservation, load shifting, and the uptake of low-emission technologies such as electric cars and residential solar panels. A widespread adoption of these behaviors represents one of the cornerstones of climate protection policies (Dietz et al., 2009; Stern et al., 2016; Ivanova et al., 2020). However, while existing laboratory and survey research can serve as a tentative guide for policy makers aiming to implement normative, pecuniary and commitment strategies in policy design, there are clear limitations of these methodologies in terms of internal and/or external validity: most notable is the difficulty of transplanting findings beyond laboratory settings and the commonly used convenience samples (Levitt and List, 2007; Mitchell, 2012; Galizzi and Navarro-Martinez, 2019), the difficulty of drawing causal conclusions from correlational research (Ferraro and Miranda, 2014, 2017; Wichman and Ferraro, 2017), as well as biased responses when behavior is self-reported rather than objectively measured (Kormos and Gifford, 2014; but see Vesely and Klöckner, 2020). All these issues are compounded by more general methodological limitations of much previous research in the social sciences, especially the reliance on small samples, leading to unreliable findings (Stanley et al., 2018). For these and other reasons, researchers in the energy domain are turning to field experiments, a method particularly well-suited for accurate program evaluation and for drawing policy recommendations (Frederiks et al., 2016).

A number of previous meta-analyses focus on the role of social norms (Delmas et al., 2013; Andor et al., 2020a; Buckley, 2020; Nemati and Penn, 2020), prices (Faruqui and Sergici, 2010; Delmas et al., 2013; Labandeira et al., 2017, 2020; Zhu et al., 2018; Buckley, 2020; Nemati and Penn, 2020) and commitment (Andor et al., 2020a) in the energy domain. Here we in part build on these efforts, reflecting their conclusions in our overall evaluation of the field. The present review is nevertheless built on a considerably broader evidence base than any of the previous meta-analyses. We also review the latest research in the area produced since the publication of previous meta-studies. By synthesizing findings from previous meta-analyses, as well as from recent studies, we are able to provide the most comprehensive overview of research on norms, incentives and commitment in the energy domain up to date.

Unlike any of the previous research syntheses, we in addition provide a detailed outline of factors possibly moderating the interventions' effects (i.e., interventions being more or less effective in some contexts or for some target groups). Current research in the energy domain increasingly focuses on the contexts and target group characteristics that may contribute to the interventions' success or failure, as this is of great theoretical but also applied interest (e.g., Andor et al., 2020b). Despite this growing interest, and as our review reveals, research on factors moderating intervention effects specifically in the energy field is still quite limited. To supplement this limited evidence, we discuss moderators that were identified in studies other than field experiments (e.g., in lab experiments) and in studies focusing on pro-environmental and sustainable behaviors more generally and not solely in the energy domain.

Thus, our goal is to evaluate the likely effects of applying three types of interventions (norms, incentives, and commitment) in the energy behavior domain, as well as discussing possible moderators of the interventions' effects (the latter discussion being based also on the broader literature on sustainable behavior). We deliberately abstain from discussing the manifold and diverse theoretical underpinnings of norm compliance (here the interested reader is referred for example to Cialdini et al., 1990; Bicchieri, 2006; Jacobson et al., 2011), commitment and goal setting (see e.g., Frederick et al., 2002; Locke and Latham, 2002), and incentive effects (see e.g., Camerer and Hogarth, 1999; Bonner and Sprinkle, 2002), as these are not directly relevant to this work's objectives.

*Social norm* interventions are based on providing participants of field experiments with information about the behavior of other people and about the socially acceptable standards of behavior. They have been employed in hundreds of previous field experiments on energy conservation (for seminal contributions see especially Schultz et al., 2007 and Allcott, 2011b). An advantage of norm-based interventions is that they are easily scalable (Allcott, 2011b; Bator et al., 2014) and often fairly cost-effective (Allcott and Rogers, 2014; Gillingham and Tsvetanov, 2018; but see Andor et al., 2020b). Behavioral effects, however, are typically only modest in magnitude, as detailed in Section Overall effectiveness of social norm interventions. We suspect this may be partly due to the treatment interacting with baseline behavior levels (Schultz et al., 2007, 2016) and other factors such as electricity prices (Sudarshan, 2017), decision observability (Vesely and Klöckner, 2018), participants' issue involvement (Göckeritz et al., 2010), group identification (De Dominicis et al., 2019) or political orientation (Costa and Kahn, 2013). We therefore discuss possible boundary conditions and moderators of social norm intervention effects in Section Boundary conditions and moderators of intervention effects.

We further discuss interventions based on providing participants in field experiments with monetary *incentives* (e.g., rebates) or with information on financial implications of their actions (e.g., financial savings that can be achieved by purchasing an energy efficient appliance). It is a standard assumption in economics that incentives shape and constrain people's decisions. Thus, it is not surprising that people can be motivated by financial considerations also in the context of energy-related behaviors, including hybrid and battery electric vehicle adoption (Hardman et al., 2017; Münzel et al., 2019), installation of residential solar panels (Dharshing, 2017; Bollinger et al., 2020), preferences for green electricity (Ek and Söderholm, 2008; Neumann and Mehlkop, 2020), and electricity consumption (Faruqui and Sergici, 2010; Labandeira et al., 2017). We take a closer look at the magnitude of incentive effects reported in previous field experimental studies in Section Effectiveness of incentive-based interventions overall.

In *commitment/goal-setting* interventions, participants are asked to commit to future behaviors or specific behavioral goals that are related to reducing energy consumption. They have been used, with varying degrees of success, to encourage various sustainable behaviors, including unplugging electrical appliances when not in use (van der Werff et al., 2019), electricity conservation (Pallak and Cummings, 1976; Loock et al., 2013), water conservation (Jaeger and Schultz, 2017), travel mode choice (Matthies et al., 2006), and towel reuse during hotel stays (Baca-Motes et al., 2013; Terrier and Marfaing, 2015). Meta-analyses of experimental studies by Abrahamse and Steg (2013), Lokhorst et al. (2013) and Nisa et al. (2019) indicate that commitment tends to have a positive effect on pro-environmental behavior, but as we show in Section Effectiveness of commitment-based interventions overall, the usefulness of commitment-based strategies in the energy behavior domain appears to be limited.

The focus of our paper is on social norms, incentives and commitment. In selecting these three intervention types, we cover the prime price and non-price intervention approaches in the energy behavior field, while keeping the scope of the review manageable. Specifically, we chose to exclude feedback interventions from our review (the interested reader is referred e.g., to Karlin et al., 2015) and information-based interventions. Additionally, we also chose not to cover interventions that have only been deployed occasionally so far and including them in a review may be premature before additional evidence accumulates (e.g., competition or pro-social incentives, see Alberts et al., 2016; Azarova et al., 2020).

Unlike most previous research syntheses, we pay particularly close attention to potential moderators of the interventions' effects. Moderators are factors that can render the effect of an intervention more or less pronounced (MacKinnon, 2011; Hayes, 2013). For instance, when an intervention motivates behavioral change in one segment of the population (e.g., people above a certain age) but leaves another segment of the population unaffected (e.g., people below a certain age), then the characteristic that defines this population segment (i.e., age) is expected to moderate the intervention's effect.

We deem moderators to be highly relevant from an applied perspective: Suppose a field trial shows an intervention having a small overall effect on energy conservation (a very common outcome, as we shall see in the Results Section). This can mean different things depending on the presence of influential moderators. Assuming that there are no variables moderating the intervention's effect, the intervention has a small effect on all its targets across the board, and as a result, the policy maker may decide to discard it from future programs due to its lack of effectiveness. In a second scenario, let us assume that there is a variable strongly moderating the intervention's effect – e.g., young people do not respond to it at all and older people respond to it strongly. In this second scenario, it makes sense for the policy maker to target subgroups responsive to the treatment in subsequent deployments of the intervention program.

## Methods

### Inclusion criteria

The following criteria were applied to select studies for inclusion in the part of the literature review focusing on the interventions' main effects (i.e., Sections Overall effectiveness of social norm interventions, Effectiveness of incentive-based interventions overall, and Effectiveness of commitment-based interventions overall below):

(1) The study was a published or unpublished empirical study (e.g., journal article, book chapter, working paper) or a meta-analysis of empirical studies. The full text of the study was accessible through our database subscriptions.
(2) The method used in the study was either a field experiment or a quantitative meta-analysis of field experimental studies.
(3a) Among the treatments investigated in the study was at least one of the following: social norms, incentives, commitment.
(4a) Among the dependent variables was at least one behavior in the energy domain (e.g., energy consumption, adoption of energy efficient appliances).
(5) If a study was included in one of the meta-analyses covered in the present review, that study itself was not included in the present review in order to minimize bias due to double-counting of effect sizes.

For inclusion of studies in the part of the literature review focusing on moderators of the interventions' effects, we retained the above criterion (1), omitted criteria (2) and (5), and relaxed criteria (3a) and (4a) as follows: ^1^

(3b) Among the (measured or manipulated) independent variables investigated in the study was at least one of the following: social norms, incentives, commitment.
(4b) Among the dependent variables was at least one pro-environmental behavior (e.g., energy consumption, adoption of energy efficient appliances, recycling).^2^

Finally, to be included in the part of the review focusing on moderators, the study had to:

Present results for one or more moderators of the effect of one or more of the independent variables norms, incentives, and commitment.

### Literature search and selection of studies

We located sources potentially relevant for inclusion in our review using three search strategies:

(1) The first strategy consisted of searching the Web of Science database using a combination of terms such as “norm,” “social comparison” and “field experiment.” The search string is reproduced in Appendix. This way, we located 31,052 potentially relevant sources.
(2) The second strategy consisted of ancestry and descendancy searches. This yielded 83 additional potentially relevant sources.
(3) Finally, we included 23 additional potentially relevant papers previously known to the authors.

The search was completed in March 2021. In the next step, we screened the abstracts of all sources located *via* the above search strategies, retaining those that could not be excluded based on the inclusion criteria presented in Section Inclusion criteria. This resulted in a selection of 429 potentially relevant sources. The full texts of these sources were then inspected to determine whether they met our inclusion criteria. Fifty studies met criteria for inclusion in the sections on main effects, 101 studies met criteria for inclusion in the sections on moderator effects, and 26 sources met both sets of criteria.

### Coding of studies

For each source included in the review, we coded the methodology used, the target behavior, the type of intervention, an approximate size of the effect of the intervention (see the next paragraph for details), and whether there were relevant moderator effects.

For the purposes of this report, a change of < 5 percent compared to control was considered a “small” effect, a change of 5–10 percent was considered a “medium-sized” effect, and a change of more than 10 percent was considered a “large” effect. Similarly, a change of 40 percent or less of standard deviation compared to control was considered a small effect, a change of 40–80 percent of standard deviation was considered a medium-sized effect, and a change of over 80 percent of standard deviation was considered a large effect. A correlation below |0.2| was considered a small effect, a correlation between |0.2| and |0.4| was considered medium-sized, and a correlation above |0.4| was considered large.

These cut-offs are selected based on established recommendations (e.g., Cohen, 1988) and taking into account opinions of scholars and practitioners in the energy field. In case an effect reported in a primary study or meta-analysis was statistically indistinguishable from zero, we state that there was “no effect” (rather than that the effect was “small”). Only the direction of the effect is coded when its size cannot be determined from what is reported in the primary study. It should be noted that it is not always easy to compare the effect size metrics used in the different papers (e.g., percentage change vs. change expressed in standard deviation units). The above interpretation of the quantitative values is therefore only meant to give an approximate sense of the quantitative data, without attempting to precisely compare the effect sizes across studies.

**Figure 1:**
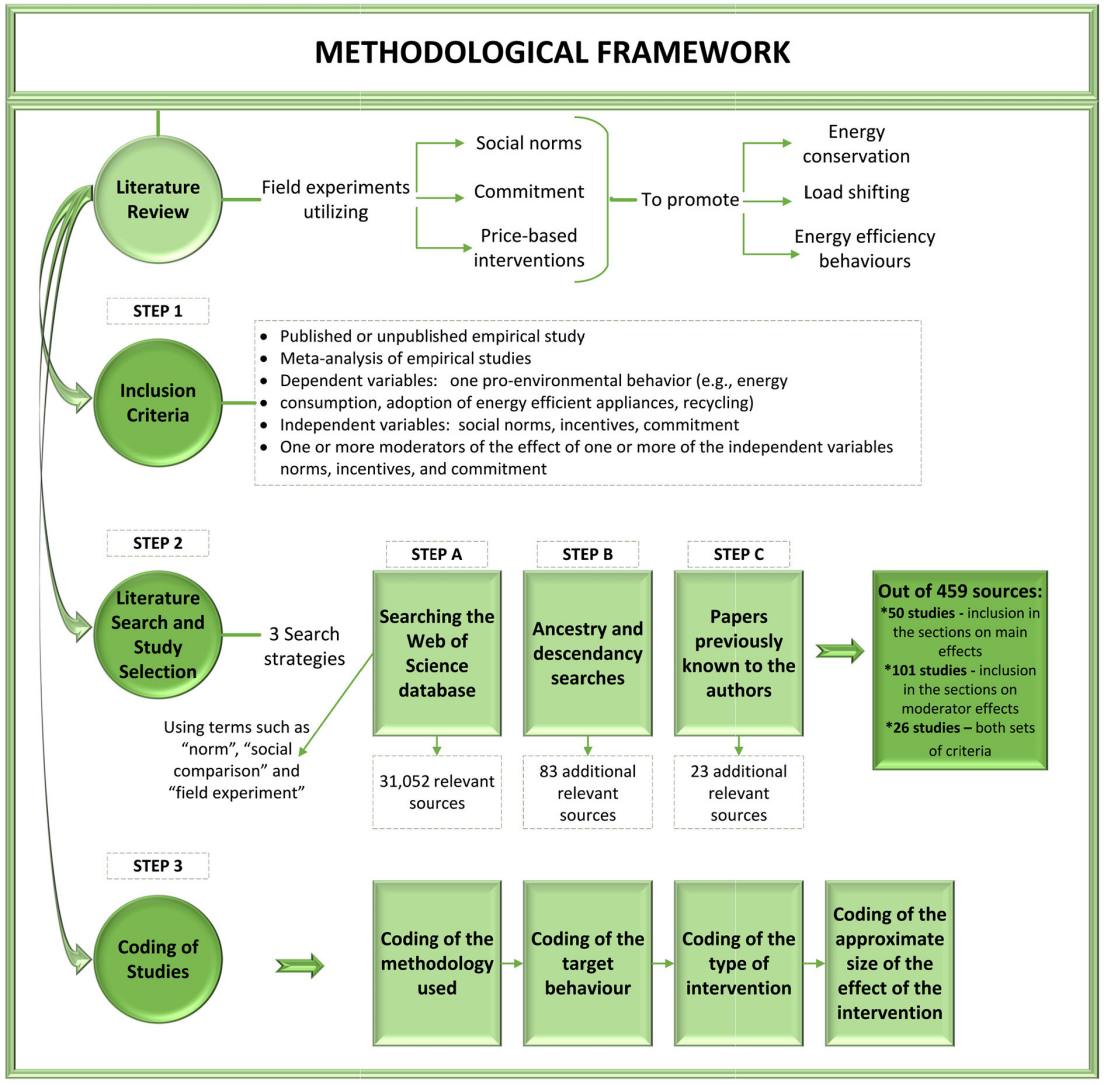
Methodological framework.

We summarize the methodological framework used in our paper in Figure 1.

## Results

For each intervention, we first present an overview of the results regarding their overall effect, then review possible moderators and boundary conditions of those effects, and conclude by discussing limitations of existing research and directions for future research.

### Social norms interventions

#### Overall effectiveness of social norm interventions

As documented in Table 1, previous social norm field experiments mostly achieved small and often statistically insignificant reductions in energy consumption (e.g., Delmas et al., 2013; Jachimowicz et al., 2018; Buckley, 2020). However, larger effects were occasionally observed (e.g., Leoniak and Cwalina, 2019; Brülisauer et al., 2020). Furthermore, observational and survey research suggests the possibility of substantial norm effects on eco-friendly technology adoption (Graziano and Gillingham, 2015; Barth et al., 2016; Wolske et al., 2017; Bollinger et al., 2022). However, there are almost no field experiments that leverage social norms to promote eco-friendly technology adoption—with the exception of Beltramo et al. (2015, finding no effect of norms on adoption of fuel efficient cook stoves in Uganda), Bollinger et al. (2022, finding no effect of norms on adoption of fuel efficient cook stoves in Mali), Bollinger et al. (2020, finding a large effect on the adoption of residential solar panels), and partly Holladay et al. (2019, finding no effect on energy efficiency investments *via* normative messages promoting home energy audits).

**Table 1:** Overview of former research—social norms.

**Source**	**Main methodology used**	**Main target behavior(s)**	**Effect of social norm confirmed?**
Andor et al. (2020a)	Meta-analysis of field experimental studies	Energy conservation	Small decrease in energy consumption
Buckley (2020)	Meta-analysis of field experimental studies	Energy conservation	No effect (meta-regression results, full model specification)
Delmas et al. (2013)	Meta-analysis of field experimental studies	Energy conservation	No effect (meta-regression results, full model specification)
Jachimowicz et al. (2018)	Meta-analysis of field experimental studies	Energy conservation	Small decrease in energy consumption (results for Opower trials)
Andor et al. (2020b)	Field experiment	Energy conservation	Small, marginally significant decrease in energy consumption (the estimate reaches conventional levels of statistical significance in models with outliers removed)
Bator et al. (2019)	Field experiment	Energy conservation	Small short-term and moderate long-term decrease in energy consumption (results for the “feedback” treatment in Study 2)
Beltramo et al. (2015)	Field experiment	Adoption of fuel-efficient cook stoves	No effect
Bogard et al. (2020)	Field experiment	Energy conservation	Moderate decrease to small increase (sic!) in energy consumption (depending on treatment)
Bollinger et al. (2020)	Field experiment	Adoption of residential solar panels	Large increase in installations relative to control (result for the “pro-social” treatment; longer-term post-campaign effects not considered here)
Bonan et al. (2019)	Field experiment	Energy conservation	No effect
Bonan et al. (2021)	Field experiment	Adoption of fuel-efficient cook stoves	No effect
Bonan et al. (2020)	Field experiment	Energy conservation	No effect to small decrease in energy consumption (depending on program duration)
Brandon et al. (2017)	Field experiment	Energy conservation	Small decrease in energy consumption
Brandon et al. (2019)	Field experiment	Energy conservation during peak-load events	Small to moderate decrease in energy consumption (depending on treatment)
Brülisauer et al. (2020)	Field experiment	Energy conservation	Large decrease in energy consumption
Byrne et al. (2018)	Field experiment	Energy conservation	No effect
Caballero and DellaValle (2021)	Field experiment	Energy conservation	Mixed results depending on model specification (no effect in the model without controls; moderate decrease in consumption in the model with psycho-social controls; large increase in consumption in the model with household and demographic controls)
Charlier et al. (2021)	Field experiment	Energy conservation	No effect
Crago et al. (2020)	Field experiment	Energy conservation	No effect
Henry et al. (2019)	Field experiment	Energy conservation	Small decrease in energy consumption
Holladay et al. (2019)	Field experiment	Takeup of home energy audits	No effect (result for social comparisons in terms of CO_2_ emissions) Large increase in audit takeup (results for social comparisons in terms of energy consumption and in terms of energy consumption expenditures)
		Investment in home energy efficiency improvements	No effect
Kácha and Ruggeri (2019)	Field experiment	Energy conservation	No effect
Kandul et al. (2020)	Field experiment	Energy conservation	Small decrease in temperature setting
Komatsu and Nishio (2015)	Field experiment	Motivation to conserve energy	No effect to small increase in motivation compared to control (depending on the type of normative message used)
Leoniak and Cwalina (2019)	Field experiment	Switching off unused lights	Large increase in light switching (main effect of the injunctive norm sign compared to request only in Studies 1 and 2) No effect (main effect of the descriptive norm sign compared to request only in Studies 1 and 2)
List et al. (2017)	Field experiment	Energy conservation	Small decrease in energy consumption
Liu et al. (2016)	Field experiment	Signing a petition to adjust a public building's thermostat to save energy	Moderate increase in likelihood of signing the petition
Mi et al. (2020a)	Field experiment	Energy conservation	No effect (result for the “normative information” condition)
Mi et al. (2020b)	Field experiment	Energy conservation	Large decrease in energy consumption (result for groups with comparative feedback)
Murakami et al. (2022)	Field experiment	Energy conservation during peak-demand hours	No effect
Myers and Souza (2020)	Field experiment	Energy conservation	No effect
Ojima et al. (2019)	Field experiment	Energy conservation	No effect (compared to a feedback only condition)
Pellerano et al. (2017)	Field experiment	Energy conservation	Small decrease in energy consumption
Wong-Parodi et al. (2019)	Field experiment	Energy conservation	Large decrease in energy consumption

#### Boundary conditions and moderators of intervention effects

To answer the question whether social norm interventions are more effective under select conditions and when specific subgroups are targeted, we provide an overview of possible boundary conditions and moderators of social norm intervention effects proposed and tested in the literature. Here, we emphasize that the evidence base concerning factors modulating the effectiveness of social norms is still limited. Thus, the findings presented below, should be regarded as tentative prior to additional replication efforts (see Maniadis et al., 2014; Allcott, 2015).

We find that there are 14 possible moderators of social norm effects, which we discuss in turn in the following:

(a) Baseline behavior levels. Participants with higher energy consumption at baseline tend to be more responsive to social norm information (e.g., Allcott, 2011b; Ayres et al., 2013; Ferraro and Price, 2013; Byrne et al., 2018; Andor et al., 2020b; Brülisauer et al., 2020; but see Schultz et al., 2015; Henry et al., 2019). This is especially true when high pre-treatment users also endorse pro-environmental values (Bonan et al., 2019).
(b) Group identification. People who identify with a reference group are more likely to adhere to that group's norms. For evidence from an energy conservation field experiment, see De Dominicis et al. (2019). Dixon et al. (2015), on the other hand, found no evidence for a moderating effect of group identification in their survey on energy conservation in the workplace. Thus, while the notion of an interaction between group norms and group identification is relatively uncontroversial in other sustainability domains (Terry et al., 1999; Fielding et al., 2008; White et al., 2009; Masson and Fritsche, 2014; Bertoldo and Castro, 2016; for related evidence on the role of “self-construal” see White and Simpson, 2013), only tenuous support for this hypothesis currently exists in the context of performing energy-related behaviors.
(c) Proximity of the norm source. Existing research indicates that proximity of the norm source (the reference group) matters for norm-compliance in the context of energy and resource conservation (Goldstein et al., 2008; Loock et al., 2012; Shen et al., 2016) and eco-friendly technology adoption (Graziano and Gillingham, 2015; Barth et al., 2016; Bonan et al., 2021; Bollinger et al., 2022). At least up to a point, greater proximity of the reference group seems to be associated with its greater normative influence (but see Mertens and Schultz, 2021). Among the reasons for a greater influence of (relatively) proximal reference groups could be that people are more aware of them (Bollinger et al., 2022), that the conduct of these groups provides cues that seem more pertinent to the decision maker's own situation (Goldstein et al., 2008; Passafaro et al., 2019) or that the decision maker identifies with these groups (Agerström et al., 2016).
(d) Subjective social norms. Participants with strong subjective social norms for energy conservation at baseline are more responsive to normative interventions (Anderson et al., 2017).
(e) Personal norms. Participants holding strong personal pro-environmental norms are less susceptible to social norms conveyed *via* interventions (Schultz et al., 2016; but see Wan et al., 2017).
(f) Issue involvement. Somewhat similar to the previous case, participants exhibiting greater personal involvement in conservation issues are less responsive to social norms (Göckeritz et al., 2010; see also Lapinski et al., 2017).
(g) Environmental concern. Environmental concern does not seem to reliably predict how people respond to normative peer influence (Moons and De Pelsmacker, 2012, 2015; Delmas and Lessem, 2014). Results reported in Ek and Söderholm (2008), however, suggest that people scoring high on environmental concern may be more receptive to social norms in the context of purchasing electricity from renewable sources.
(h) Innovativeness. Innovative individuals do not seem to differ from others in their willingness to align their behavior with perceived normative expectations of their peers (Moons and De Pelsmacker, 2015; Lundheim et al., 2021).
(i) Altruism. Delmas and Lessem (2014) report that conformity with energy saving norms is generally not affected by one's altruism.
(j) Decision observability. People can become more norm compliant when their decisions are publicly observable—see Vesely and Klöckner (2018) for an experiment on donations to environmental organizations, and Babutsidze and Chai (2018) for a correlational study focusing on a range of pro-environmental behaviors. However, this effect was not confirmed in the context of investments in renewable energy (Vesely et al., 2022). More broadly, Nemati and Penn (2020) report that when behavior was publicly observable, information-based interventions (including, but not limited to, norm-based interventions) had more pronounced effects on electricity conservation.
(k) Behavior difficulty and other costs. Sudarshan (2017) report that an intervention utilizing normative feedback led to reduced consumption of cheap grid electricity, but not of more expensive electricity generated from a backup diesel source. Thus, when prices were high, norms seemed powerless to motivate energy conservation.
  Andersson and von Borgstede (2010), on the other hand, found that while perceived social norms influenced both low-cost and high-cost waste recycling behaviors in households, normative influences were more pronounced in the latter. In another study on household recycling, however, Hage et al. (2009) found no evidence for an interaction between perceived social norms and a proxy for behavior difficulty, namely the access to nearby waste collection points.
  Taken together, these findings suggest that normative influences can be relatively ineffective both when behavior costs are high and when they are low, but do not offer clear answers as to when to expect which. More research in this area is needed, especially in the context of energy-related decisions.
(l) Cultural context. Analyses reported by Bergquist et al. (2019) suggest that people in more individualistic countries (e.g., many European countries) may be more responsive to pro-environmental social norms than people in collectivistic countries (e.g., many Asian countries, see Hofstede et al., 2010). However, this finding should be interpreted with caution, as it is based on a meta-analysis across studies with design differences, which were not taken into account in the analysis. A counter-example can be found in a questionnaire study by Eom et al. (2016), where social norms predicted intention to purchase eco-friendly products in participants from a highly collectivist country (Japan), but not in participants from a highly individualistic country (the US).
  Cultural differences (broadly speaking) exist within nations as well—some suggestive evidence on how these can impact the effectiveness of norm-based interventions is provided by Gillingham and Tsvetanov (2018). They detected a substantially greater effect of their intervention, designed to promote the uptake of home energy audits, in rural compared to urban areas (but see Loock et al., 2012 reporting comparable norm effects in urban and rural areas).
(m) Perceived social norms appear to shape moral obligation to engage in energy conservation and efficiency behaviors only in liberals, but not in conservatives, in the United States (Arpan et al., 2013). Costa and Kahn (2013) report heterogeneous treatment effects that can be traced back to political ideology, with liberals responding more strongly than conservatives to energy conservation norms. No differences in treatment effects related to the political ideology of the intervention's targets were, however, found in Gillingham and Tsvetanov (2018). Using municipality-level data from Germany, Inhoffen et al. (2019) detected weaker peer effects on solar panel installations in municipalities with larger Green party vote shares (however, this result should be interpreted as suggestive, due to the spatial data aggregation).

#### Limitations of existing research and future directions

We see four main areas in which subsequent research can advance our understanding of social norm effects, as well as policy applications of norm-based interventions.

First, it would be beneficial to broaden the scope of targeted behaviors. Previous social norm field experiments focused primarily on low-cost curtailment behaviors, notably on encouraging people to curtail their energy consumption at home and in public spaces (Allcott, 2011b; Bator et al., 2014; Leoniak and Cwalina, 2019) and on other simple low-cost, low-involvement actions like towel reuse (Goldstein et al., 2008; Schultz et al., 2008), tire pressure checks (Yeomans and Herberich, 2014) and closing windows when the heating is on (Ornaghi et al., 2018). Even though a portion of energy use reductions achieved in energy conservation campaigns à la Allcott (2011b) seems to stem from energy efficiency investments, rather than solely from a change in habits (Brandon et al., 2017), promoting household energy efficiency and eco-friendly investments also more directly would be useful.

With the exception of Beltramo et al. (2015), Holladay et al. (2019), Bollinger et al. (2020) and Bonan et al. (2021), we are aware of no previous field experiments using norm-based interventions to motivate high-cost investment decisions in the energy domain, such as purchasing electric cars or solar panels. This contrasts with an abundance of correlational studies on eco-friendly technology adoption (e.g., Korcaj et al., 2015; Barth et al., 2016; Wolske et al., 2017; Noppers et al., 2019). Future field experimental research should devote increased attention to a wider variety of energy-related choices, comprising both curtailment and investment. Motivating such a broader range of high-cost, high-involvement behaviors is essential for achieving ambitious climate change mitigation goals (Dietz et al., 2009; Stern et al., 2016; Bjelle et al., 2018; Ivanova et al., 2020).

Results reported by McCoy and Lyons (2017) even suggest that interventions (in their case, providing feedback *via* smart meters) successfully targeting energy curtailment may unintentionally inhibit households' investments in energy efficiency, supporting the argument for targeting both investment and curtailment behaviors.

Secondly, replicating promising findings on factors modulating interventions' effects is needed. Previous research suggests that social norm interventions can be particularly effective, for example, when targeting intensive energy users, or those with strong ties to their (norm) reference group, as discussed in Section Boundary conditions and moderators of intervention effects. However, initially promising findings need to be replicated and tested in field conditions.

Third, subsequent research should carefully evaluate possible unintended side effects of normative interventions. One limitation of earlier studies is that the interventions' impact on their targets' emotional wellbeing has been largely neglected. Interventions are typically solely evaluated in terms of their effects on the target behavior, and sometimes in terms of their cost-effectiveness. Evidence on how social norm interventions designed to foster sustainable energy-related behavior affect their targets' emotional wellbeing is sparse and inconclusive. A number of studies suggest that social norm interventions can induce negative emotional states, for example anger (Aronson and O'Leary, 1982-83; Allcott, 2011b; Sussman and Gifford, 2012; Ayres et al., 2013; Costa and Kahn, 2013; Bergquist and Nilsson, 2016), other studies suggest a positive effect on emotions (Delmas and Lessem, 2014; Vesely et al., 2022), and some studies detect no discernible effects or report mixed findings (Toner et al., 2014; Allcott and Kessler, 2019; Leoniak and Cwalina, 2019). Ensuring that customers' and constituents' emotional discomfort does not result from an intervention is vital for companies and policy makers. Such considerations are similarly vital in terms of promoting subsequent sustainable behaviors; in particular, Carrus et al. (2021) specifically support the role of emotions and their strong link with energy-related behaviors and intentions in their meta-analysis of previous research on this topic.

Finally, to understand if social norm interventions work, it is necessary to isolate their unique effects (i.e., effects that can be uniquely attributed to norms and not to other intervention elements). Many “social norm” interventions employed in field experiments on energy conservation combine information on social norms with additional treatment elements. For example, in Allcott (2011b), Ayres et al. (2013), Costa and Kahn (2013) and many other studies, social norm information is augmented with feedback on own consumption and with energy saving advice. Bator et al. (2019) and Mack et al. (2019) combine social norm information with other intervention modules, including energy saving recommendations, individual feedback, and commitment (see also Andor and Fels, 2018, who present a graphical overview of different intervention combinations in a larger set of studies). Bundling social norm information with other instruments in this way creates an identification problem, making it difficult to isolate the unique effect of norms. Thus, there is a need for more field experiments capable of estimating the unique effect of social norms (see Delmas et al., 2013; Harries et al., 2013; Anderson et al., 2017; Bhanot, 2021).

**Figure A1:**
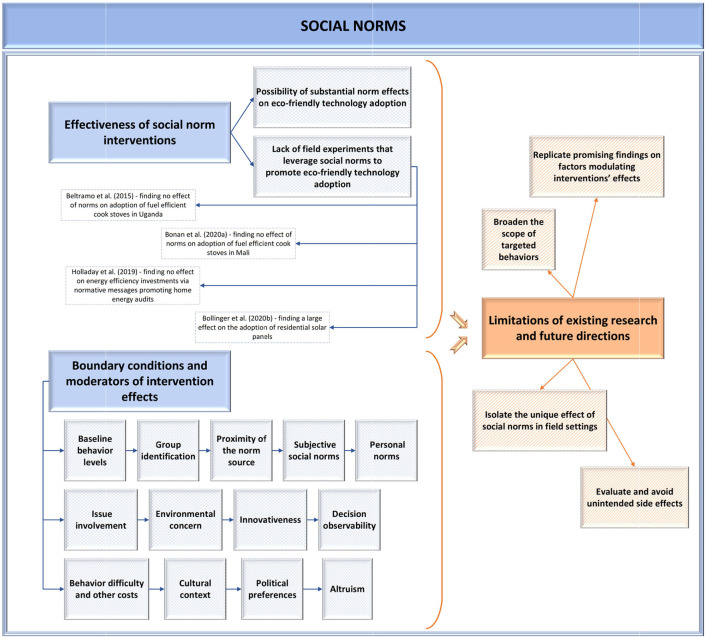
Overview of findings on social norms.

In Figure A1 in the Appendix, we summarize the results of the analysis of social norm interventions.

### Incentive-based interventions

#### Effectiveness of incentive-based interventions overall

Incentive-based interventions typically achieve small or statistically insignificant reductions in energy use, as documented in Supplementary Table 1 (e.g., Delmas et al., 2013; Buckley, 2020). However, cases of larger effects are not unknown (Faruqui and Sergici, 2010; Ito et al., 2018; Burkhardt et al., 2019; Bollinger and Hartmann, 2020). In the next section, we discuss a number of factors potentially contributing to the interventions' success or failure.

Concerning eco-friendly technology adoption, most field experiments found no effect of pecuniary strategies (e.g., Allcott and Sweeney, 2017; Gillingham and Bollinger, 2021). However, interestingly, large effects have been found in a number of cases (e.g., Allcott and Taubinsky, 2015; Gillan, 2018; Bollinger et al., 2020; Fowlie et al., 2021), and some non-experimental studies also suggest a substantial potential of monetary instruments (see Gallagher and Muehlegger, 2011; Münzel et al., 2019). For a detailed overview see Supplementary Table 1.

Incentives seem to represent a potentially powerful policy instrument, despite the real possibility that an incentive-based strategy will be unsuccessful. Thus, there is a need for continued examination of the types of incentive-based strategies that fare better than others, and of the specific contexts and recipient characteristics affecting their performance. For example, cash incentives may be more effective than providing information on prospective savings in some cases (Allcott and Taubinsky, 2015; Rodemeier and Löschel, 2020).

Moreover, even when incentives have the desired effect initially, research in a number of domains, including energy conservation and eco-friendly technology adoption, suggests that the effects may quickly dissipate once incentives are withdrawn (Dharshing, 2017; Ito et al., 2018; Azarova et al., 2020). Many authors also argue that incentives partially crowd out intrinsic motivation and pro-social motives (Deci, 1971; Deci et al., 1999; but see Steinhorst and Klöckner, 2018; Kaiser et al., 2020). Thus, if the mere provision of incentives crowds out intrinsic motivation and pro-sociality to some extent, incentives need to be powerful enough to create a positive net effect on the desired outcome.

Another question concerns the relative suitability of positive motivation (e.g., subsidies and discounts) vs. negative motivation (e.g., taxes and fines). As far as behavioral outcomes go, negative motivation may be somewhat more effective (for evidence from a labor context see Hossain and List, 2012; Hong et al., 2015). Research on public acceptance of sustainability policies, however, indicates that the more heavy-handed “push” measures (e.g., taxes and fines) are typically less acceptable than softer “pull” measures like subsidies and rebates (Tobler et al., 2012; Rhodes et al., 2017; Mahmoodi et al., 2018; Keizer et al., 2019).

#### Boundary conditions and moderators of intervention effects

Are incentive-based interventions more effective under certain conditions and when targeting specific subgroups of the population? Apart from studies exploring the role of socio-demographic moderators, existing research in the energy-behavior domain is largely silent on this issue. Given the paucity of available data, we should therefore once again underscore that the findings presented here should be regarded as tentative prior to several rounds of successful replication.

Our literature review suggests eight possible moderators of incentive-based effects, which are discussed below.

(a) Socio-demographic characteristics. Numerous studies^3^ provide evidence that socio-demographic characteristics, including age, gender, education, income, home ownership, and parental status, help determine people's responsiveness to incentives and prices. Findings are, however, typically mixed and inconclusive. For example, lower-income consumers sometimes appear to be more responsive to incentives and energy and technology prices (Reiss and White, 2005; Alberini et al., 2011; Allcott, 2011a; Ito, 2015; Ida et al., 2016; DeShazo et al., 2017; Houde, 2018; Charlier and Kahouli, 2019; Lundgren and Schultzberg, 2019; Schmitz and Madlener, 2020), but other studies disconfirm or qualify this link (Nesbakken, 1999; Herter, 2007; Faruqui et al., 2013; Moshiri, 2015; Zhang, 2015; Schulte and Heindl, 2017; Hansen, 2018; Alberini et al., 2019; Harding et al., 2020; Prest, 2020). Inconsistent findings could be partly due to methodological differences across studies or context-dependency of the effects.
(b) Political preferences and environmental concern. Schwartz et al. (2015) and Xu et al. (2015) report that the relative effectiveness of monetary vs. environmental appeals depends on the target audience's political preferences (and environmental concern, in Xu et al., 2015).
(c) Personal norms. Steinhorst and Matthies (2016) found that people with strong (but not people with weak) pro-environmental personal norms may be more responsive to information about the negative environmental impacts of energy consumption compared to information about monetary savings associated with energy conservation. Hunecke et al. (2001), on the other hand, found no interaction between incentives and personal norms in the context of travel mode choice. For related evidence concerning the role of personal norms and values in the context of paper conservation appeals, see van den Broek et al. (2017).
(d) Environmental identity and attitudes. DellaValle and Zubaryeva (2019) indicate that individuals scoring high on pro-environmental identity may be more responsive to incentive-based interventions promoting the uptake of eco-friendly technology. See also Fenrick et al. (2014) for suggestive evidence of environmental attitudes affecting consumer responses to electricity pricing.
(e) Time preferences. Response to potential future cost-savings from eco-friendly technology adoption appears to be steeper for individuals more willing to delay consumption (DellaValle and Zubaryeva, 2019).
(f) Baseline behavior levels. Findings concerning responsiveness to energy prices of households with different consumption levels are mixed (Herter, 2007; Kaza, 2010; Gilbert and Graff Zivin, 2014; List et al., 2017; Royal and Rustamov, 2018; Harding et al., 2020; Prest, 2020; Schmitz and Madlener, 2020; Todd-Blick et al., 2020; Murakami et al., 2022). This could be partly due to context- or behavior-specificity of the effects (see Kaza, 2010). In addition, it may be difficult to detect subtle non-linear effects with conventional methods (see Prest, 2020).
(g) Context effects. The influence of incentives on energy-related behaviors may depend on the context determined by other policies and intervention tools, for example information provision (see Hayes and Cone, 1977; Sexton et al., 1989; Allcott, 2011a; Ashraf et al., 2013; Kahn and Wolak, 2013; Krause et al., 2013; Harding and Lamarche, 2016; Jenn et al., 2018; Palmer et al., 2018; Figueroa et al., 2019; Bollinger and Hartmann, 2020; Frondel and Kussel, 2020; Harding et al., 2020; Rodemeier and Löschel, 2020; McKenna et al., 2021), feedback on own consumption (see Hayes and Cone, 1977; Woo et al., 2013; Jessoe and Rapson, 2014; Harding and Lamarche, 2016; Martin and Rivers, 2018; Bollinger and Hartmann, 2020; Harding et al., 2020; Prest, 2020; McKenna et al., 2021), social norms feedback (see Dolan and Metcalfe, 2015; List et al., 2017; Sudarshan, 2017; Rezvani et al., 2018; Brent and Wichman, 2020), or the use of enabling and automation technologies like smart programmable thermostats (see Faruqui and Sergici, 2011; Davis et al., 2013; Faruqui et al., 2013; Suter and Shammin, 2013; Woo et al., 2013; Harding and Lamarche, 2016; Gillan, 2018; Bollinger and Hartmann, 2020; Harding et al., 2020).

#### Limitations of existing research and future directions

Three main avenues for subsequent research are apparent. As in the case of social norm interventions, replicating promising findings on factors modulating interventions' effects is necessary before drawing firm theoretical and policy conclusions. Economic theory provides substantial support for the general idea that the effects of monetary incentives vary according to individuals, and that contextual factors modulate incentive effects. This issue should be more thoroughly investigated in the domain of energy-related behavior, building on research by Reiss and White (2005), Schwartz et al. (2015), Xu et al. (2015), and others.

Secondly, the presence of incentives may alter the effect of interventions invoking social norms, pro-environmental attitudes and other intrinsic motives (Drews et al., 2020; but see Brent and Wichman, 2020; West et al., 2021). Results reported by Sudarshan (2017) showcase the potential importance of this issue for energy conservation campaigns. The author found that, in the absence of monetary incentives to conserve energy, adding incentives to a norm- and feedback-based intervention completely eliminated the positive effect of the intervention. Pellerano et al. (2017) report a similar finding. The usefulness of complementing financial incentives with non-pecuniary instruments (and vice versa) thus needs to be assessed in subsequent research.

And finally, one may wonder about the long-term effects of incentives on behavior and motivation. It can be useful to examine whether, and if so, to what extent, their effect remains after incentives are discontinued (see e.g., Ito et al., 2018; Azarova et al., 2020), and to what extent provision of incentives affects intrinsic motivation (see Steinhorst and Klöckner, 2018).

**Figure A2:**
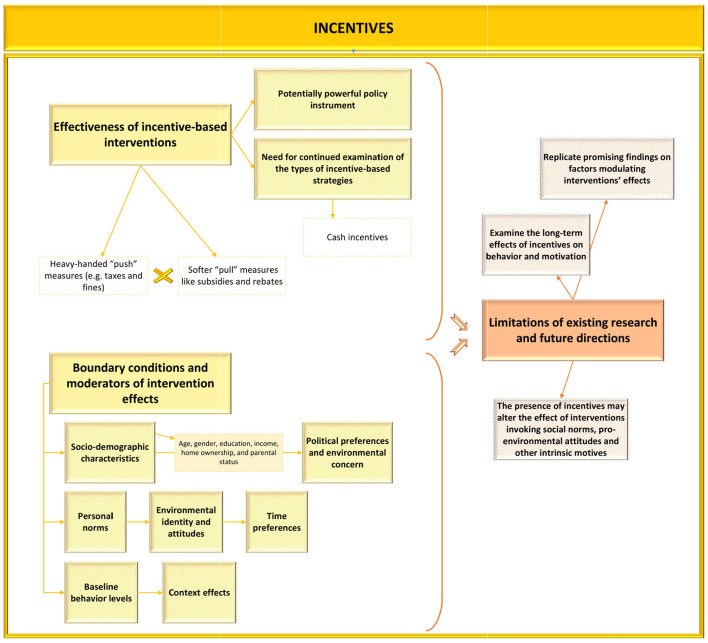
Overview of findings on incentives.

The results of our analysis of incentive-based interventions are illustrated in Figure A2 in the Appendix.

### Commitment and goal setting

#### Effectiveness of commitment-based interventions overall

As documented in Table 2, the effectiveness of commitment and goal setting strategies employed in previous field experimental studies on energy conservation has been very limited (e.g., Lokhorst et al., 2015; van der Werff et al., 2019; Andor et al., 2020a; but see Aydin et al., 2018 who found suggestive evidence for a strong effect of combining feedback and goal setting in their field quasi-experiment). The results are slightly more encouraging for pro-environmental behavior more generally (Lokhorst et al., 2013; Nisa et al., 2019). A possible approach therefore is to increase the salience of the environmental impact of intense energy consumption when asking participants to commit to energy conservation goals.

**Table 2:** Overview of former research—commitment and goal setting.

**Source**	**Main methodology used**	**Main target behavior(s)**	**Effect of commitment confirmed?**
Andor et al. (2020a)	Meta-analysis of field experimental studies	Energy conservation	No effect (results for self-set goals)
			Small decrease in energy consumption (results for externally set goals)
Barata et al. (2017)	Field experiment	Energy conservation	No effect
Bell et al. (2016)	Field experiment	Energy conservation	Moderate increase in self-reported energy conservation behaviors
Ghesla et al. (2020)	Field experiment	Energy conservation	No effect (result for the “goal” treatment)
Legault et al. (2020)	Field experiment	Energy conservation	No effect (result for the motivational and goal-setting intervention)
Lokhorst et al. (2015)	Field experiment	Energy conservation	No effect on most behaviors, but a large increase in room temperature setting (i.e., an increase in energy consumption)
Loock et al. (2013)	Field experiment	Energy conservation	No effect to small decrease in energy consumption (depending on treatment)
Löschel et al. (2020)	Field experiment	Energy conservation	No effect
Shen et al. (2019)	Field experiment	Energy conservation	No effect
van der Werff et al. (2019)	Field experiment	Completely switching off unused appliances (instead of using standby)	No effect

#### Boundary conditions and moderators of intervention effects

Evidence concerning possible boundary conditions and moderators of the effect of commitment on energy-related behaviors is extremely limited. What evidence there is suggests that the following six factors may moderate the success of commitment interventions:

(a) Personal pro-environmental norms. Matthies et al. (2006) demonstrate that the efficacy of commitment-based strategies is enhanced when the committing individuals hold strong personal norms in favor of the target behavior.
(b) Values. People holding strong “egoistic” values appear to be more responsive to commitment opportunities, and people with strong “biospheric” values are sometimes less responsive (Brandsma and Blasch, 2019).
(c) Public commitment. Commitments made in public appear to be more effective than private commitments (Pallak and Cummings, 1976; see also Epton et al., 2017). People may also be more willing to commit when doing so publicly rather than privately (see Exley and Naecker, 2017 for evidence from an academic context).
(d) Behavior difficulty. van der Werff et al. (2019) report that commitment can be used as a lever to motivate difficult energy-saving behaviors, but not easy behaviors.
(e) Goal difficulty. When combined with feedback on own consumption, difficult energy saving goals appear to be more effective in curbing consumption than easier ones (Becker, 1978; for related evidence from other domains see Epton et al., 2017).
(f) Feedback provision. When difficult energy saving goals are set, feedback provision boosts energy conservation. Feedback does not appear to facilitate conservation when easy to achieve energy saving goals are set (Becker, 1978; for related evidence from other domains see Neubert, 1998; Epton et al., 2017).

#### Limitations of existing research and future directions

Additional research is needed to examine the emotional experiences that accompany commitment. These would presumably depend on whether or not a commitment was made, and, if so, on whether or not the actor successfully completed the behavior or goal to which they committed. The possibility of negative emotions that may be associated especially with public commitment is discussed in Lokhorst et al. (2015), and results in Löschel et al. (2020) illustrate that people may prefer to avoid receiving goal-setting nudges. On the other hand, Baca-Motes et al. (2013) and Joo et al. (2018) found no negative effects of commitment interventions on customer satisfaction in the context of resource conservation campaigns in hotels.

As we found only very limited evidence regarding influential moderators of commitment strategies, subsequent research is called for investigating the attitudinal, personality and contextual moderators affecting their success. Null results reported e.g., in Lokhorst et al. (2015) stress the need for a careful implementation of commitment-based strategies, including the consideration of influential moderators of the interventions' effects.

Similarly to the case of social norm research, also in case of studies on commitment it would be useful to isolate the unique effect of commitment and goal setting interventions. One problem with existing research is that commitment-based interventions are oftentimes coupled with other intervention elements, such as energy consumption feedback and energy saving tips (Abrahamse et al., 2007; Harding and Hsiaw, 2014; Mack et al., 2019; Mi et al., 2019; Legault et al., 2020), which precludes a clean attribution of the interventions' effects specifically to commitment.

Finally, to the best of our knowledge, although relatively easy to implement, there are no field experimental studies on the influence of prior commitment on subsequent eco-friendly technology adoption and energy-efficiency investments. This is a promising area for future investigations.

**Figure A3:**
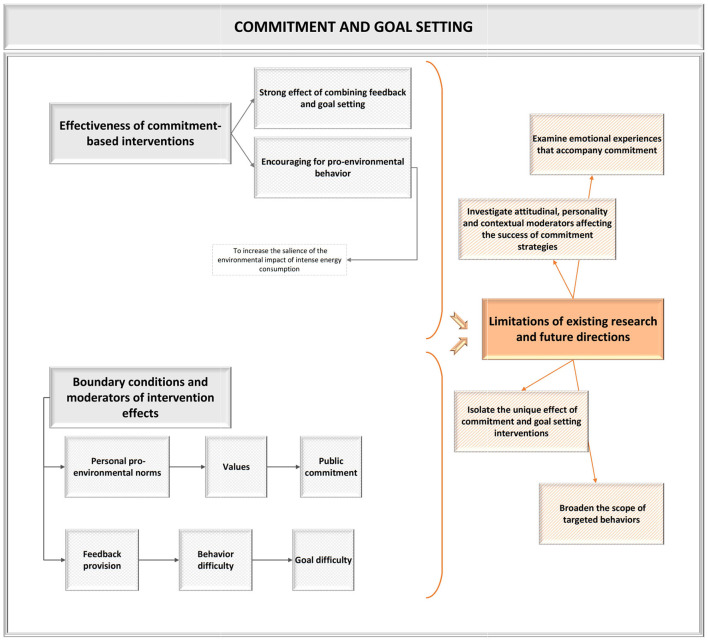
Overview of findings on commitment.

Figure A3 in the Appendix provides a graphical presentation of the key results presented in this subsection about commitment and goal setting interventions.

## Concluding discussion

**Figure 2:**
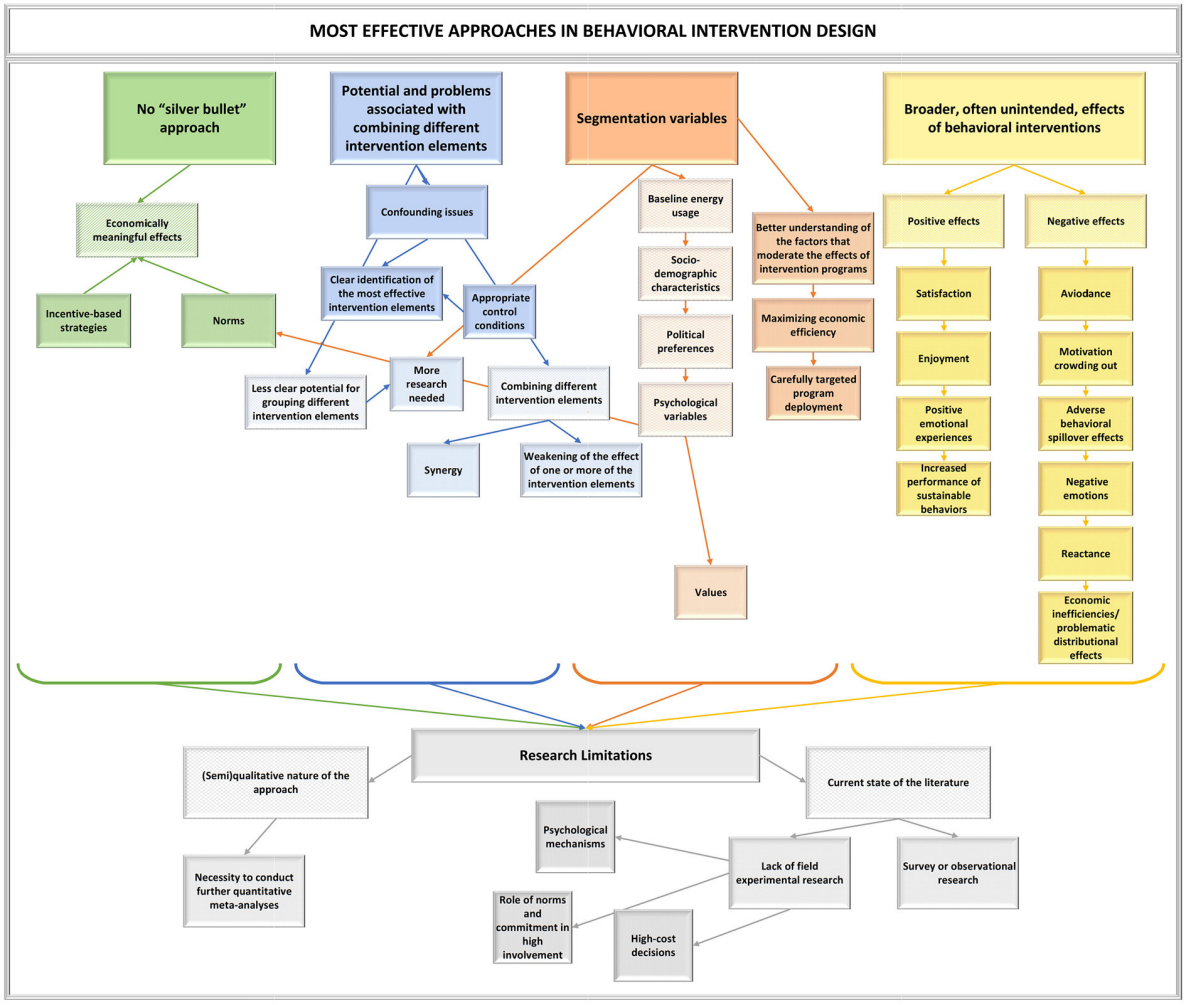
Overview of general conclusions.

This literature review gives us an idea of what could be the most effective approaches in behavioral intervention design. In Figure 2, we present a graphical summary of the key outcomes and results of our analysis, which we discuss below.

First, we show that there is no “silver bullet” approach. None of the intervention types have reliably large effects (i.e., irrespective of the context and target population). However, some interventions seem to have greater potential than others. In a number of cases, economically meaningful effects have been achieved with incentive-based strategies (e.g., Faruqui and Sergici, 2010; Allcott and Taubinsky, 2015; Ito et al., 2018; Bollinger et al., 2020) and norms (e.g., Leoniak and Cwalina, 2019; Brülisauer et al., 2020). The evidence to support the usefulness of commitment and goal setting strategies is much weaker.

Second, we discussed the potential, as well as the problems associated with combining different intervention elements (e.g., norms and incentives) in a single intervention package. The drawbacks are clear: confounding issues preclude a clear identification of the most effective intervention elements when they are bundled without appropriate control conditions (cf. Delmas et al., 2013; Harries et al., 2013; Frederiks et al., 2016; Bhanot, 2021). The potential for grouping different intervention elements is less clear, and this important issue requires more research. As discussed in a recent paper by Drews et al. (2020), combining different intervention elements can lead to synergy (the intervention package as a whole having a stronger effect than each individual component), but it can also lead to a weakening of the effect of one or more of the intervention elements. While intuition suggests that interventions with more elements are more effective (cf. Abrahamse et al., 2007; Dietz et al., 2009; Osbaldiston and Schott, 2012; Mack et al., 2019), research often rather suggests an absence of synergistic effects when using multiple intervention elements (Harries et al., 2013; Schwartz et al., 2015; Alberts et al., 2016; Anderson et al., 2017; Pellerano et al., 2017; Sudarshan, 2017; Martin and Rivers, 2018).

Third, regardless of whether the intervention has a generally relatively strong effect, the effect can be substantially increased by targeting the intervention to the most receptive groups. The segmentation variables that we reviewed include, for example, baseline energy usage (Schultz et al., 2007, 2016; Allcott, 2011b), socio-demographic characteristics (Bollinger et al., 2020; Rodemeier and Löschel, 2020; Stojanovski et al., 2020), political preferences (Costa and Kahn, 2013; Schwartz et al., 2015; Xu et al., 2015), and psychological variables, such as personal norms (Schultz et al., 2016; Steinhorst and Matthies, 2016) and values (Bonan et al., 2019; Brandsma and Blasch, 2019). More research is needed to consolidate our understanding of the role of the various segmentation variables. A better understanding of the factors that moderate the effects of intervention programs is a prerequisite for maximizing economic efficiency *via* carefully targeted program deployment. Similarly, a shift toward more advanced, tailored and sequentially adaptive intervention programs (such as those showing promise in the medical field, see e.g., Nahum-Shani et al., 2018; Miller, 2019) depends on insights into the type of interventions most suited to particular individuals.

Fourth, researchers and practitioners' attention should focus on the broader, often unintended, effects of behavioral interventions. These can be positive, such as satisfaction, enjoyment and other positive emotional experiences (Herter, 2007; Delmas and Lessem, 2014; Vesely et al., 2022), and increased performance of sustainable behaviors not targeted by the interventions (Steinhorst et al., 2015; Carlsson et al., 2021; Jessoe et al., 2021; see Maki et al., 2019; Geiger et al., 2021 for meta-analyses). However, negative effects include avoidance (Löschel et al., 2020), motivation crowding out (Lavergne et al., 2010; Schwartz et al., 2015), adverse behavioral spillover effects (Tiefenbeck et al., 2013; McCoy and Lyons, 2017; Bjelle et al., 2018), negative emotions (Sussman and Gifford, 2012) and reactance (Bergquist and Nilsson, 2016), as well as economic inefficiencies (Allcott and Greenstone, 2012) and problematic distributional effects (Azarova et al., 2019; White and Sintov, 2020). To find the best approach, interventions thus need to be thoroughly pre-tested prior to their large-scale roll out, and evaluated not only in terms of their intended behavioral impact, but also in terms of their (unintended) downstream effects.

Like all literature reviews, we were limited by the semi-qualitative nature of our approach. As new evidence accumulates, it will be appropriate to conduct further quantitative meta-analyses covering these topics. Still, our approach made it possible to combine evidence from previous meta-analyses and more recent studies in a much more comprehensive manner than previous works in the area. To lay the groundwork for the future, experimenters should adhere to reporting standards conducive to subsequent research synthesis efforts.

Another limitation of the present study stemmed from the current state of the literature itself. In particular, our conclusions were not always based solely on field experimental research (where no or very little field experimental evidence was available). Instead, we occasionally resorted to evidence from laboratory, survey or observational research to illustrate various points. To some extent, this is perhaps inevitable, as field experimental research can only go so far when examining for example the underlying psychological mechanisms. On the other hand, in some cases, field experimental research was conspicuous by its absence, especially regarding the role of norms and commitment in high-involvement, high-cost decisions such as the uptake of eco-friendly technologies. This research gap and others outlined in this review point to many opportunities for meaningful research contributions.

## Data availability statement

The original contributions presented in the study are included in the article/supplementary files, further inquiries can be directed to the corresponding author/s.

## Author contributions

Conceptualization and writing—review and editing: SV, CK, GC, LT, FC, MB, AK, and AS. Methodology: SV, CK, GC, LT, and FC. Data collection and analysis and writing—original draft: SV. All authors contributed to the article and approved the submitted version.

## Funding

This research was supported by a grant from the EU (ENCHANT project, grant agreement no. 957115). This work was additionally supported by a grant of the Ministry of Education and Research to AS, CNCS/CCCDI-UEFISCDI, project number PN-III-P3.6-H2020-2020-0063.

## Conflict of interest

The authors declare that the research was conducted in the absence of any commercial or financial relationships that could be construed as a potential conflict of interest.

## Publisher's note

All claims expressed in this article are solely those of the authors and do not necessarily represent those of their affiliated organizations, or those of the publisher, the editors and the reviewers. Any product that may be evaluated in this article, or claim that may be made by its manufacturer, is not guaranteed or endorsed by the publisher.

## Footnotes

1 The reason for modifying the inclusion criteria for the sections focusing on moderators was the limited number of studies reporting relevant moderator effects that met the stricter criteria.

2 Note that naturally we also cite studies unrelated to pro-environmental behavior as part of discussing the main findings.

3 See e.g., Nesbakken (1999), Reiss and White (2005), Herter (2007), Rehdanz (2007), Meier and Rehdanz (2010), Alberini et al. (2011, 2019), Allcott (2011a), Mills and Schleich (2012), Ito (2015), Moshiri (2015), Yang and Zhao (2015), Zhang (2015), Bjerkan et al. (2016), Ida et al. (2016), Wichman et al. (2016), Zhang et al. (2016), DeShazo et al. (2017), Schulte and Heindl (2017), Hansen (2018), Houde (2018), Ščasný et al. (2018), Silva et al. (2018), Sovacool et al. (2018, 2019), Charlier and Kahouli (2019), Kormos et al. (2019), Lundgren and Schultzberg (2019), Bollinger et al. (2020), Deryugina et al. (2020), Harding et al. (2020), Jenn et al. (2020), Rodemeier and Löschel (2020), Schmitz and Madlener (2020), Stojanovski et al. (2020), and Wolske (2020).

## Appendix: Search string used to search the Web of Science database

TOPIC: ((norm* OR peer* OR “social comparison*” OR “social influence*” OR “social feedback” OR “social nudge*” OR “social information” OR “social pressure” OR “social signal*” OR “social group*” OR “group comparison*” OR “group feedback” OR “group pressure” OR conform* OR “home energy report*” OR “model*ing” OR “neighbo*” OR “block leader*” OR commit* OR goal* OR pledge* OR “self*control” OR “self*regulation” OR incentiv* OR pric* OR money OR pay* OR cash OR cost* OR sale* OR discount* OR expenditure* OR financ* OR monetary OR pecuniary OR economic OR subsid* OR rebate* OR tariff* OR tax* OR credit* OR reward* OR penal* OR sanction* OR gain* OR loss* OR “time*of*use” OR “critical*peak” OR “peak*demand” OR “peak*time” OR “real*time pric*” OR “electricity bill*” OR “energy bill*” OR waive*) AND (“field experiment*” OR “field stud*” OR “field trial*” OR “randomized controlled trial*” OR RCT OR pilot* OR “program* evaluation*”) AND (energy OR electric* OR renewable* OR wind OR solar OR consum* OR conserv* OR curtail* OR reduc* OR “energy saving*” OR “electricity saving*” OR “energy us*” OR “electricity us*” OR “energy demand” OR “electricity demand” OR “demand management” OR “demand response” OR invest* OR adopt* OR purchas* OR buy* OR subscri* OR uptake OR install* OR weatheariz* OR heat* OR cooling OR “air conditioning” OR energy audit* OR “electric car*” OR “electric vehicle*” OR e-car* OR hybrid* OR “electric bicycle*” OR e-bike* OR “energy*efficien*” OR “smart meter*” OR technolog* OR appliance* OR photovoltaic*)) Refined by: WEB OF SCIENCE CATEGORIES: (ENVIRONMENTAL SCIENCES OR GREEN SUSTAINABLE SCIENCE TECHNOLOGY OR PSYCHOLOGY OR ENERGY FUELS OR ENGINEERING ELECTRICAL ELECTRONIC OR TRANSPORTATION SCIENCE TECHNOLOGY OR MANAGEMENT OR EDUCATION SCIENTIFIC DISCIPLINES OR ENGINEERING ENVIRONMENTAL OR BUSINESS OR ENVIRONMENTAL STUDIES OR ECONOMICS OR OPERATIONS RESEARCH MANAGEMENT SCIENCE OR MULTIDISCIPLINARY SCIENCES OR TELECOMMUNICATIONS OR INFORMATION SCIENCE LIBRARY SCIENCE OR BEHAVIORAL SCIENCES OR ENGINEERING MULTIDISCIPLINARY OR PSYCHOLOGY APPLIED OR SOCIAL SCIENCES INTERDISCIPLINARY OR PSYCHOLOGY MULTIDISCIPLINARY OR EDUCATION EDUCATIONAL RESEARCH) Timespan: All years. Indexes: SCI-EXPANDED, SSCI, A&HCI, CPCI-S, CPCI-SSH, ESCI.

## References

[B1] Abrahamse W. Steg L. (2013). Social influence approaches to encourage resource conservation: A meta-analysis. Glob. Environ. Change 23, 1773–1785. 10.1016/j.gloenvcha.2013.07.029

[B2] Abrahamse W. Steg L. Vlek C. Rothengatter T. (2007). The effect of tailored information, goal setting, and tailored feedback on household energy use, energy-related behaviors, and behavioral antecedents. J. Environ. Psychol. 27, 265–276. 10.1016/j.jenvp.2007.08.002

[B3] Agerström J. Carlsson R. Nicklasson L. Guntell L. (2016). Using descriptive social norms to increase charitable giving: The power of local norms. J. Econ. Psychol. 52, 147–153. 10.1016/j.joep.2015.12.007

[B4] Alberini A. Gans W. Velez-Lopez D. (2011). Residential consumption of gas and electricity in the U.S.: The role of prices and income. Energy Econ. 33, 870–881. 10.1016/j.eneco.2011.01.015

[B5] Alberini A. Khymych O. Ščasný M. (2019). Response to extreme energy price changes: Evidence from Ukraine. Energy J. 40, 189–212. 10.5547/01956574.40.1.aalb

[B6] Alberts G. Gurguc Z. Koutroumpis P. Martin R. Muûls M. Napp T. (2016). Competition and norms: A self-defeating combination? Energy Policy 96, 504–523. 10.1016/j.enpol.2016.06.001

[B7] Allcott H. (2011a). Rethinking real-time electricity pricing. Resour. Energy Econ. 33, 820–842. 10.1016/j.reseneeco.2011.06.003

[B8] Allcott H. (2011b). Social norms and energy conservation. J. Public Econ. 95, 1082–1095. 10.1016/j.jpubeco.2011.03.003

[B9] Allcott H. (2015). Site selection bias in program evaluation. Q. J. Econ. 130, 1117–1165. 10.1093/qje/qjv015

[B10] Allcott H. Greenstone M. (2012). Is there an energy efficiency gap? J. Econ. Perspect. 26, 3–28. 10.1257/jep.26.1.3

[B11] Allcott H. Kessler J. B. (2019). The welfare effects of nudges: A case study of energy use social comparisons. Am. Econ. J. Appl. Econ. 11, 236–276. 10.1257/app.20170328

[B12] Allcott H. Rogers T. (2014). The short-run and long-run effects of behavioral interventions: Experimental evidence from energy conservation. Am. Econ. Rev. 104, 3003–3037. 10.1257/aer.104.10.3003

[B13] Allcott H. Sweeney R. L. (2017). The role of sales agents in information disclosure: Evidence from a field experiment. Manage. Sci. 63, 21–39. 10.1287/mnsc.2015.2327

[B14] Allcott H. Taubinsky D. (2015). Evaluating behaviorally motivated policy: Experimental evidence from the lightbulb market. Am. Econ. Rev. 105, 2501–2538. 10.1257/aer.20131564

[B15] Anderson K. Song K. Lee S. Krupka E. Lee H. Park M. (2017). Longitudinal analysis of normative energy use feedback on dormitory occupants. Appl. Energy 189, 623–639. 10.1016/j.apenergy.2016.12.086

[B16] Andersson M. von Borgstede C. (2010). Differentiation of determinants of low-cost and high-cost recycling. J. Environ. Psychol. 30, 402–408. 10.1016/j.jenvp.2010.02.003

[B17] Andor M. Bensch G. Fels K. Kneppel N. (2020a). Per Stups zum Energiesparen? Eine Meta-Analyse zu den kausalen Effekten von verhaltensökonomischen Interventionen auf den Energieverbrauch privater Haushalte. Perspekt. Wirtschaftspolitik 20, 352–382. 10.1515/pwp-2018-0039

[B18] Andor M. A. Fels K. M. (2018). Behavioral economics and energy conservation – a systematic review of nonprice interventions and their causal effects. Ecol. Econ. 148, 178–210. 10.1016/j.ecolecon.2018.01.018

[B19] Andor M. A. Gerster A. Peters J. Schmidt C. M. (2020b). Social norms and energy conservation beyond the US. J. Environ. Econ. Manage. 103, 102351. 10.1016/j.jeem.2020.102351

[B20] Aronson E. O'Leary M. (1982-83). The relative effectiveness of models and prompts on energy conservation: A field experiment in a shower room. J. Environ. Syst. 12, 219–224. 10.2190/UBD5-4Y9B-61EF-WUM6

[B21] Arpan L. M. Opel A. R. Lu J. (2013). Motivating the skeptical and unconcerned: Considering values, worldviews, and norms when planning messages encouraging energy conservation and efficiency behaviors. Appl. Environ. Educ. Communic. 12, 207–219. 10.1080/1533015X.2013.838875

[B22] Ashraf N. Jack B. K. Kamenica E. (2013). Information and subsidies: Complements or substitutes? J. Econ. Behav. Organiz. 88, 133–139. 10.1016/j.jebo.2012.12.031

[B23] Aydin E. Brounen D. Kok N. (2018). Information provision and energy consumption: Evidence from a field experiment. Energy Econ. 71, 403–410. 10.1016/j.eneco.2018.03.008

[B24] Ayres I. Raseman S. Shih A. (2013). Evidence from two large field experiments that peer comparison feedback can reduce residential energy usage. J. Law Econ. Organiz. 29, 992–1022. 10.1093/jleo/ews020

[B25] Azarova V. Cohen J. J. Kollmann A. Reichl J. (2020). Reducing household electricity consumption during evening peak demand times: Evidence from a field experiment. Energy Policy 144, 111657. 10.1016/j.enpol.2020.111657

[B26] Azarova V. Engel D. Ferner C. Kollmann A. Reichl J. (2019). Exploring the impact of network tariffs on household electricity expenditures using load profiles and socio-economic characteristics. Nat. Energy 3, 317–325. 10.1038/s41560-018-0105-4

[B27] Babutsidze Z. Chai A. (2018). Look at me saving the planet! The imitation of visible green behavior and its impact on the climate value-action gap. Ecol. Econ. 146, 290–303. 10.1016/j.ecolecon.2017.10.017

[B28] Baca-Motes K. Brown A. Gneezy A. Keenan E. A. Nelson L. D. (2013). Commitment and behavior change: Evidence from the field. Journal of Consumer Research 39, 1070–1084. 10.1086/667226

[B29] Barata R. Castro P. Martins-Loução M. A. (2017). How to promote conservation behaviours: The combined role of environmental education and commitment. Environ. Educ. Res. 23, 1322–1334. 10.1080/13504622.2016.1219317

[B30] Barth M. Jugert P. Fritsche I. (2016). Still underdetected – social norms and collective efficacy predict the acceptance of electric vehicles in Germany. Transport. Res. Part F 37, 64–77. 10.1016/j.trf.2015.11.011

[B31] Bator R. J. Phelps K. Tabanico J. Schultz P. W. Walton M. L. (2019). When it is not about the money: Social comparison and energy conservation among residents who do not pay for electricity. Energy Res. Soc. Sci. 56, 101198. 10.1016/j.erss.2019.05.00811241113

[B32] Bator R. J. Tabanico J. J. Walton M. L. Schultz P. W. (2014). Promoting energy conservation with implied norms and explicit messages. Soc. Influence 9, 69–82. 10.1080/15534510.2013.778213

[B33] Becker L. J. (1978). Joint effect of feedback and goal setting on performance: FIELD-study of residential energy-conservation. J. Appl. Psychol. 63, 428–433. 10.1037/0021-9010.63.4.428

[B34] Bell B. T. Toth N. Little L. Smith M. A. (2016). Planning to save the planet: Using an online intervention based on implementation intentions to change adolescent self-reported energy-saving behavior. Environ. Behav. 48, 1049–1072. 10.1177/0013916515583550

[B35] Beltramo T. Blalock G. Levine D. I. Simons A. M. (2015). Does peer use influence adoption of efficient cookstoves? Evidence from a randomized controlled trial in Uganda. J. Health Communic. 20, 55–66. 10.1080/10810730.2014.994244 25839203

[B36] Bergquist M. Nilsson A. (2016). I saw the sign: Promoting energy conservation via normative prompts. J. Environ. Psychol. 46, 23–31. 10.1016/j.jenvp.2016.03.005

[B37] Bergquist M. Nilsson A. Schultz W. P. (2019). A meta-analysis of field-experiments using social norms to promote pro-environmental behaviors. Glob. Environ. Change 59, 101941. 10.1016/j.gloenvcha.2019.101941

[B38] Bertoldo R. Castro P. (2016). The outer influence inside us: Exploring the relation between social and personal norms. Resour. Conserv. Recycl. 112, 45–53. 10.1016/j.resconrec.2016.03.020

[B39] Bhanot S. P. (2021). Isolating the effect of injunctive norms on conservation behavior: New evidence from a field experiment in California. Organiz. Behav. Hum. Decis. Processes. 163, 30–42. 10.1016/j.obhdp.2018.11.002

[B40] Bicchieri C. (2006). The Grammar of Society: The Nature and Dynamics of Social Norms. Cambridge: Cambridge University Press. 10.1017/CBO9780511616037

[B41] Bjelle E. L. Steen-Olsen K. Wood R. (2018). Climate change mitigation potential of Norwegian households and the rebound effect. J. Clean. Prod. 172, 208–217. 10.1016/j.jclepro.2017.10.089

[B42] Bjerkan K. Y. Nørbech T. E. Nordtømme M. E. (2016). Incentives for promoting Battery Electric Vehicle (BEV) adoption in Norway. Transport. Res. Part D 43, 169–180. 10.1016/j.trd.2015.12.002

[B43] Bogard J. E. Delmas M. A. Goldstein N. J. Vezich I. S. (2020). Target, distance, and valence: Unpacking the effects of normative feedback. Organ. Behav. Hum. Decis. Process. 161, 61–73. 10.1016/j.obhdp.2020.10.003

[B44] Bollinger B. Gillingham K. Kirkpatrick A. J. Sexton S. (2022). Visibility and peer influence in durable good adoption. Market. Sci. 41, 453–476. 10.1287/mksc.2021.1306

[B45] Bollinger B. Gillingham K. T. Ovaere M. (2020). Field experimental evidence shows that self-interest attracts more sunlight. Proc. Nat. Acad. Sci. U. S. A. 117, 20503–20510. 10.1073/pnas.2004428117 32778577 PMC7456121

[B46] Bollinger B. K. Hartmann W. R. (2020). Information vs. automation and implications for dynamic pricing. Manag. Sci. 66, 290–314. 10.1287/mnsc.2018.3225

[B47] Bonan J. Battiston P. Bleck J. LeMay-Boucher P. Pareglio S. Sarr B. . (2021). Social interaction and technology adoption: Experimental evidence from improved cookstoves in Mali. World Dev. 144, 105467. 10.1016/j.worlddev.2021.105467

[B48] Bonan J. Cattaneo C. d'Adda G. Tavoni M. (2019). Heterogeneity of Social Information Programs: The Role of Identity and Values. Working paper.

[B49] Bonan J. Cattaneo C. d'Adda G. Tavoni M. (2020). The interaction of descriptive and injunctive social norms in promoting energy conservation. Nat. Energy 5, 900–909. 10.1038/s41560-020-00719-z

[B50] Bonner S. E. Sprinkle G. B. (2002). The effects of monetary incentives on effort and task performance: Theories, evidence, and a framework for research. Account. Organiz. Soc. 27, 303–345. 10.1016/S0361-3682(01)00052-6

[B51] Brandon A. Ferraro P. J. List J. A. Metcalfe R. D. Price M. K. Rundhammer F. (2017). Do the Effects of Social Nudges Persist? Theory and Evidence From 38 Natural Field Experiments. Working paper. 10.3386/w23277

[B52] Brandon A. List J. A. Metcalfe R. D. Price M. K. Rundhammer F. (2019). Testing for crowd out in social nudges: Evidence from a natural field experiment in the market for electricity. Proc. Nat. Acad. Sci. U. S. A. 116, 5293–5298. 10.1073/pnas.1802874115 30104369 PMC6431171

[B53] Brandsma J. S. Blasch J. E. (2019). One for all? – the impact of different types of energy feedback and goal setting on individuals' motivation to conserve electricity. Energy Policy 135, 110992. 10.1016/j.enpol.2019.110992

[B54] Brent D. A. Wichman C. J. (2020). Do Behavioral Nudges Interact With Prevailing Economic Incentives? Pairing Experimental and Quasi-Experimental Evidence from Water Consumption. Working paper.

[B55] Brülisauer M. Goette L. Jiang Z. Schmitz J. Schubert R. (2020). Appliance-specific feedback and social comparisons: Evidence from a field experiment on energy conservation. Energy Policy 145, 111742. 10.1016/j.enpol.2020.111742

[B56] Buckley P. (2020). Prices, information and nudges for residential electricity conservation: A meta-analysis. Ecol. Econ. 172, 106635. 10.1016/j.ecolecon.2020.106635

[B57] Burkhardt J. Gillingham K. Kopalle P. K. (2019). Experimental Evidence on the Effect of Information and Pricing on Residential Electricity Consumption. Working paper. 10.3386/w25576

[B58] Byrne D. P. La Nauze A. Martin L. A. (2018). Tell me something I don't already know: Informedness and the impact of information programs. Rev. Econ. Stat. 100, 510–527. 10.1162/rest_a_00695

[B59] Caballero N. Della Valle N. (2021). Tackling energy poverty through behavioral change: A pilot study on social comparison interventions in social housing districts. Front. Sustain. Cities 2, 601095. 10.3389/frsc.2020.601095

[B60] Camerer C. Hogarth R. M. (1999). The effects of financial incentives in experiments: A review and capital-labor-production framework. J. Risk Uncertain. 19, 7–42. 10.1023/A:1007850605129

[B61] Carlsson F. Jaime M. Villegas C. (2021). Behavioral spillover effects from a social information campaign. J. Environ. Econ. Manag. 109, 102325. 10.1016/j.jeem.2020.102325

[B62] Carrus G. Tiberio L. Mastandrea S. Chokrai P. Fritsche I. Klöckner C. A. . (2021). Psychological predictors of energy saving behaviour: A meta-analytic approach. Front. Psycholo. 12, 648221. 10.3389/fpsyg.2021.648221 34248747 PMC8265205

[B63] Charlier C. Guerassimoff G. Kirakozian A. Selosse S. (2021). Under pressure! Nudging electricity consumption within firms. Feedback from a field experiment. Energy J. 42, 129–154. 10.5547/01956574.42.1.ccha

[B64] Charlier D. Kahouli S. (2019). From residential energy demand to fuel poverty: Income-induced non-linearities in the reactions of households to energy price fluctuations. Energy J. 40, 101–138. 10.5547/01956574.40.2.dcha

[B65] Cialdini R. B. Reno R. Kallgren C. (1990). A focus theory of normative conduct: Recycling the concept of norms to reduce littering in public places. J. Pers. Soc. Psychol. 58, 1015–1026. 10.1037/0022-3514.58.6.1015

[B66] Cohen J. (1988). Statistical Power Analysis for the Behavioral Sciences, 2nd Edn. Hillsdale, NJ: Erlbaum.

[B67] Costa D. L. Kahn M. E. (2013). Energy conservation “nudges” and environmentalist ideology: evidence from a randomized residential electricity field experiment. J. Eur. Econ. Assoc. 11, 680–702. 10.1111/jeea.12011

[B68] Crago C. L. Spraggon J. M. Hunter E. (2020). Motivating non-ratepaying households with feedback and social nudges: A cautionary tale. Energy Policy 145, 111764. 10.1016/j.enpol.2020.111764

[B69] Davis A. L. Krishnamurti T. Fischhoff B. de Bruin W. B. (2013). Setting a standard for electricity pilot studies. Energy Policy 62, 401–409. 10.1016/j.enpol.2013.07.093

[B70] De Dominicis S. Sokoloski R. Jaeger C. Schultz P. W. (2019). Making the smart meter social promotes long-term energy conservation. Palgrave Communic. 5, 51. 10.1057/s41599-019-0254-5

[B71] Deci E. L. (1971). Effects of externally mediated rewards on intrinsic motivation. J. Pers. Soc. Psychol. 18, 105–115. 10.1037/h0030644

[B72] Deci E. L. Koestner R. Ryan R. M. (1999). A meta-analytic review of experiments examining the effects of extrinsic rewards on intrinsic motivation. Psychol. Bull. 125, 627–668. 10.1037/0033-2909.125.6.627 10589297

[B73] DellaValle N. Zubaryeva A. (2019). Can we hope for a collective shift in electric vehicle adoption? Testing salience and norm-based interventions in South Tyrol, Italy. Energy Res. Soc. Sci. 55, 46–61. 10.1016/j.erss.2019.05.005

[B74] Delmas M. A. Fischlein M. Asensio O. I. (2013). Information strategies and energy conservation behavior: A meta-analysis of experimental studies from 1975-2011. Energy Policy 61, 729–739. 10.1016/j.enpol.2013.05.109

[B75] Delmas M. A. Lessem N. (2014). Saving power to conserve your reputation? The effectiveness of private versus public information. J. Environ. Econ. Manag. 67, 353–370. 10.1016/j.jeem.2013.12.009

[B76] Deryugina T. MacKay A. Reif J. (2020). The long-run dynamics of electricity demand: Evidence from municipal aggregation. Am. Econ. J. Appl. Econ. 12, 86–114. 10.1257/app.20180256

[B77] DeShazo J. R. Sheldon T. L. Carson R. T. (2017). Designing policy incentives for cleaner technologies: Lessons from California's plug-in electric vehicle rebate program. J. Environ. Econ. Manag. 84, 18–43. 10.1016/j.jeem.2017.01.002

[B78] Dharshing S. (2017). Household dynamics of technology adoption: A spatial econometric analysis of residential solar photovoltaic (PV) systems in Germany. Energy Res. Soc. Sci. 23, 113–124. 10.1016/j.erss.2016.10.012

[B79] Dietz T. Gardner G. T. Gilligan J. Stern P. C. Vandenbergh M. P. (2009). Household actions can provide a behavioral wedge to rapidly reduce US carbon emissions. Proc. Nat. Acad. Sci. 106, 18452–18456. 10.1073/pnas.0908738106 19858494 PMC2767367

[B80] Dixon G. N. Deline M. B. McComas K. Chambliss L. Hoffmann M. (2015). Saving energy at the workplace: The salience of behavioral antecedents and sense of community. Energy Res. Soc. Sci. 6, 121–127. 10.1016/j.erss.2015.01.004

[B81] Dolan P. Metcalfe R. (2015). Neighbors, Knowledge, and Nuggets: Two Natural Field Experiments on the Role of Incentives on Energy Conservation. Working paper. 10.2139/ssrn.2589269

[B82] Drews S. Exadaktylos F. van den Bergh J. C. J. M. (2020). Assessing synergy of incentives and nudges in the energy policy mix. Energy Policy 144, 111605. 10.1016/j.enpol.2020.111605

[B83] Ek K. Söderholm P. (2008). Norms and economic motivation in the Swedish green electricity market. Ecol. Econ. 58, 169–182. 10.1016/j.ecolecon.2008.02.013

[B84] Eom K. Kim H. S. Sherman D. K. Ishii K. (2016). Cultural variability in the link between environmental concern and support for environmental action. Psychol. Sci. 27, 1331–1339. 10.1177/0956797616660078 27565535

[B85] Epton T. Currie S. Armitage C. J. (2017). Unique effects of setting goals on behavior change: Systematic review and meta-analysis. J. Consult. Clin. Psychol. 85, 1182–1198. 10.1037/ccp0000260 29189034

[B86] Exley C. L. Naecker J. K. (2017). Observability increases the demand for commitment devices. Manage. Sci. 63, 3262–3267. 10.1287/mnsc.2016.2501

[B87] Faruqui A. Sergici S. (2010). Household response to dynamic pricing of electricity: A survey of 15 experiments. J. Regul. Econ. 38, 193–225. 10.1007/s11149-010-9127-y

[B88] Faruqui A. Sergici S. (2011). Dynamic pricing of electricity in the mid-Atlantic region: Econometric results from the Baltimore gas and electric company experiment. J. Regul. Econ. 40, 82–109. 10.1007/s11149-011-9152-5

[B89] Faruqui A. Sergici S. Akaba L. (2013). Dynamic pricing of electricity for residential customers: The evidence from Michigan. Energy Effic. 6, 571–584. 10.1007/s12053-013-9192-z

[B90] Fenrick S. A. Getachew L. Ivanov C. Smith J. (2014). Demand impact of a critical peak pricing program: opt-in and opt-out options, green attitudes and other customer characteristics. Energy J. 35, 1–24. 10.5547/01956574.35.3.1

[B91] Ferraro P. J. Miranda J. J. (2014). The performance of non-experimental designs in the evaluation of environmental programs: A design-replication study using a large-scale randomized experiment as a benchmark. J. Econ. Behav. Organiz. 107, 344–365. 10.1016/j.jebo.2014.03.008

[B92] Ferraro P. J. Miranda J. J. (2017). Panel data designs and estimators as substitutes for randomized controlled trials in the evaluation of public programs. J. Assoc. Environ. Resour. Economists 4, 281–317. 10.1086/689868

[B93] Ferraro P. J. Price M. K. (2013). Using nonpecuniary strategies to influence behavior: Evidence from a large-scale field experiment. Rev. Econ. Stat. 95, 64–73. 10.1162/REST_a_00344

[B94] Fielding K. S. Terry D. J. Masser B. M. Hogg M. A. (2008). Integrating social identity theory and the theory of planned behaviour to explain decisions to engage in sustainable agricultural practices. Br. J. Soc. Psychol. 47, 23–48. 10.1348/014466607X206792 17535461

[B95] Figueroa A. de Moliere L. Pegels A. Never B. Kutzner F. (2019). Show me (more than) the money! Assessing the social and psychological dimensions to energy efficient lighting in Kenya. Energy Res. Soc. Sci. 47, 224–232. 10.1016/j.erss.2018.10.002

[B96] Fowlie M. Wolfram C. Baylis P. Spurlock C. A. Todd-Blick A. Cappers P. (2021). Default effects and follow-on behaviour: Evidence from an electricity pricing program. Rev. Econ. Stud. 88, 2886–2934. 10.1093/restud/rdab018

[B97] Frederick S. Loewenstein G. O'Donoghue T. (2002). Time discounting and time preference: a critical review. J. Econ. Lit. 40, 351–401. 10.1257/jel.40.2.35126063653

[B98] Frederiks E. R. Stenner K. Hobman E. V. Fischle M. (2016). Evaluating energy behavior change programs using randomized controlled trials: Best practice guidelines for policymakers. Energy Res. Soc. Sci. 22, 147–164. 10.1016/j.erss.2016.08.020

[B99] Frondel M. Kussel G. (2020). Switching on electricity demand response: Evidence for German households. Energy J. 40, 1–16. 10.5547/01956574.40.5.mfro

[B100] Galizzi M. M. Navarro-Martinez D. (2019). On the external validity of social preference games: A systematic lab-field study. Manage. Sci. 65, 976–1002. 10.1287/mnsc.2017.2908

[B101] Gallagher K. S. Muehlegger E. (2011). Giving green to get green? Incentives and consumer adoption of hybrid vehicle technology. J. Environ. Econ. Manag. 61, 1–15. 10.1016/j.jeem.2010.05.004

[B102] Geiger S. J. Brick C. Nalborczyk L. Jostmann N. B. (2021). More green than gray? Toward a sustainable overview of environmental spillover effects: A Bayesian meta-analysis. Manuscript under review. 10.31234/osf.io/u24tx

[B103] Ghesla C. Grieder M. Schmitz J. Stadelmann M. (2020). Pro-environmental incentives and loss aversion: A field experiment on electricity saving behavior. Energy Policy 137, 111131. 10.1016/j.enpol.2019.111131

[B104] Gilbert B. Graff Zivin J. (2014). Dynamic salience with intermittent billing: evidence from smart electricity meters. J. Econ. Behav. Organiz. 107, 176–190. 10.1016/j.jebo.2014.03.011

[B105] Gillan J. M. (2018). Dynamic Pricing, Attention, and Automation: Evidence from a Field Experiment in Electricity Consumption. Working paper.

[B106] Gillingham K. Bollinger B. (2021). Social learning and solar photovoltaic adoption. Manag. Sci. 67, 6629–7289. 10.1287/mnsc.2020.3840

[B107] Gillingham K. Tsvetanov T. (2018). Nudging energy efficiency audits: Evidence from a field experiment. J. Environ. Econ. Manage. 90, 303–316. 10.1016/j.jeem.2018.06.009

[B108] Göckeritz S. Schultz P. W. Rendon T. Cialdini R. B. Goldstein N. J. Griskevicius V. (2010). Descriptive normative beliefs and conservation behavior: The moderating roles of personal involvement and injunctive normative beliefs. Eur. J. Soc. Psychol. 40, 514–523. 10.1002/ejsp.643

[B109] Goldstein N. J. Cialdini R. B. Griskevicius V. (2008). A room with a view point: Using social norms to motivate environmental conservation in hotels. J. Consumer Res. 35, 472–482. 10.1086/586910

[B110] Graziano M. Gillingham K. (2015). Spatial patterns of solar photovoltaic system adoption: The influence of neighbors and the built environment. J. Econ. Geogr. 15, 815–839. 10.1093/jeg/lbu036

[B111] Hage O. Söderholm P. Berglund C. (2009). Norms and economic motivation in household recycling: Empirical evidence from Sweden. Resour. Conserv. Recycl. 53, 155–165. 10.1016/j.resconrec.2008.11.003

[B112] Hansen A. R. (2018). Heating homes: Understanding The Impact Of Prices. Energy Policy 121, 138–151. 10.1016/J.Enpol.2018.06.021

[B113] Harding M. Hsiaw A. (2014). Goal setting and energy conservation. J. Econ. Behav. Organiz. 107, 209–227. 10.1016/j.jebo.2014.04.012

[B114] Harding M. Lamarche C. (2016). Empowering consumers through data and smart technology: Experimental evidence on the consequences of time-of-use electricity pricing policies. J. Policy Anal. Manag. 35, 906–931. 10.1002/pam.21928

[B115] Harding M. Lamarche C. Pesaran M. H. (2020). Common correlated effects estimation of heterogeneous dynamic panel quantile regression models. J. Appl. Econometr. 35, 294–314. 10.1002/jae.2753

[B116] Hardman S. Chandan A. Tal G. Turrentine T. (2017). The effectiveness of financial purchase incentives for battery electric vehicles – a review of the evidence. Renew. Sust. Energy Rev. 80, 1100–1111. 10.1016/j.rser.2017.05.255

[B117] Harries T. Rettie R. Studley M. Burchell K. Chambers S. (2013). Is social norms marketing effective? A case study in domestic electricity consumption. Eur. J. Market. 47, 1458–1475. 10.1108/EJM-10-2011-0568

[B118] Hayes A. F. (2013). Introduction to Mediation, Moderation, and Conditional Process Analysis: A Regression-Based Approach. New York, NY: Guilford Press.

[B119] Hayes S. C. Cone J. D. (1977). Reducing residential electrical energy use: Payments, information, and feedback. J. Appl. Behav. Anal. 103, 425–435. 10.1901/jaba.1977.10-425 16795563 PMC1311206

[B120] Henry M. L. Ferraro P. J. Kontoleon A. (2019). The behavioural effect of electronic home energy reports: Evidence from a randomised field trial in the United States. Energy Policy 132, 1256–1261. 10.1016/j.enpol.2019.06.039

[B121] Herter K. (2007). Residential implementation of critical-peak pricing of electricity. Energy Policy 35, 2121–2130. 10.1016/j.enpol.2006.06.019

[B122] Hofstede G. Hofstede G. J. Minkov M. (2010). Cultures and Organizations: Software of the Mind, 3rd Edn. New York, NY: McGraw-Hill.

[B123] Holladay S. LaRiviere J. Novgorodsky D. Price M. (2019). Prices versus nudges: What matters for search versus purchase of energy investments? J. Public Econ. 172, 151–173. 10.1016/j.jpubeco.2018.12.004

[B124] Hong F. Hossain T. List J. A. (2015). Framing manipulations in contests: A natural field experiment. Journal of Economic Behavior and Organization 118, 372–382. 10.1016/j.jebo.2015.02.014

[B125] Hossain T. List J. A. (2012). The behavioralist visits the factory: Increasing productivity using simple framing manipulations. Manage. Sci. 58, 2151–2167. 10.1287/mnsc.1120.1544

[B126] Houde S. (2018). How consumers respond to product certification and the value of energy information. RAND J. Econ. 49, 453–477. 10.1111/1756-2171.12231

[B127] Hunecke M. Blöbaum A. Matthies E. Höger R. (2001). Responsibility and environment: Ecological norm orientation and external factors in the domain of travel mode choice behavior. Environ. Behav. 33, 830–852. 10.1177/00139160121973269

[B128] Ida T. Murakami K. Tanaka M. (2016). Electricity demand response in Japan: Experimental evidence from a residential photovoltaic power generation system. Econ. Energy Environ. Policy 5, 73–88. 10.5547/2160-5890.5.1.itak

[B129] Inhoffen J. Siemroth C. Zahn P. (2019). Minimum prices and social interactions: Evidence from the German renewable energy program. Energy Econ. 78, 350–364. 10.1016/j.eneco.2018.11.034

[B130] Ito K. (2015). Asymmetric incentives in subsidies: Evidence from a large-scale electricity rebate program. Am. Econ. J. Econ. Policy 7, 209–237. 10.1257/pol.20130397

[B131] Ito K. Ida T. Tanaka M. (2018). Moral suasion and economic incentives: Field experimental evidence from energy demand. Am. Econ. J. Econ. Policy 10, 240–267. 10.1257/pol.20160093

[B132] Ivanova D. Barrett J. Wiedenhofer D. Macura B. Callaghan M. Creutzig F. (2020). Quantifying the potential for climate change mitigation of consumption options. Environ. Res. Lett. 15, 093001. 10.1088/1748-9326/ab858928926024

[B133] Jachimowicz J. M. Hauser O. P. O'Brien J. D. Sherman E. Galinsky A. D. (2018). The critical role of second-order normative beliefs in predicting energy conservation. Nat. Hum. Behav. 2, 757–764. 10.1038/s41562-018-0434-0 31406290

[B134] Jacobson R. P. Mortensen C. R. Cialdini R. B. (2011). Bodies obliged and unbound: differentiated response tendencies for injunctive and descriptive social norms. J. Pers. Soc. Psychol. 100, 433–448. 10.1037/a0021470 21171790

[B135] Jaeger C. M. Schultz P. W. (2017). Coupling social norms and commitments: Testing the underdetected nature of social influence. J. Environ. Psychol. 51, 199–208. 10.1016/j.jenvp.2017.03.015

[B136] Jenn A. Lee J. H. Hardman S. Tal G. (2020). An in-depth examination of electric vehicle incentives: Consumer heterogeneity and changing response over time. Transport. Res. Part A 132, 97–109. 10.1016/j.tra.2019.11.004

[B137] Jenn A. Springel K. Gopal A. R. (2018). Effectiveness of electric vehicle incentives in the United States. Energy Policy 119, 349–356. 10.1016/j.enpol.2018.04.065

[B138] Jessoe K. Lade G. E. Loge F. Spang E. (2021). Spillovers from behavioral interventions: Experimental evidence from water and energy use. J. Assoc. Environ. Resour. Econ. 8, 315–346. 10.1086/711025

[B139] Jessoe K. Rapson D. (2014). Knowledge is (less) power: Experimental evidence from residential energy use. Am. Econ. Rev. 104, 1417–1438. 10.1257/aer.104.4.1417

[B140] Joo H. H. Lee J. Park S. (2018). Every drop counts: A water conservation experiment with hotel guests. Econ. Inq. 56, 1788–1808. 10.1111/ecin.12563

[B141] Kácha O. Ruggeri K. (2019). Nudging intrinsic motivation in environmental risk and social policy. J. Risk Res. 22, 581–592. 10.1080/13669877.2018.1459799

[B142] Kahn M. E. Wolak F. A. (2013). Using Information to Improve the Effectiveness of Nonlinear Pricing: Evidence From a Field Experiment. Working paper.

[B143] Kaiser F. G. Henn L. Marschke B. (2020). Financial rewards for long-term environmental protection. J. Environ. Psychol. 68, 101411. 10.1016/j.jenvp.2020.101411

[B144] Kandul S. Lang G. Lanz B. (2020). Social comparison and energy conservation in a collective action context: A field experiment. Econ. Lett. 188, 108947. 10.1016/j.econlet.2020.108947

[B145] Karlin B. Zinger J. F. Ford R. (2015). The effects of feedback on energy conservation: A meta-analysis. Psychol. Bull. 141, 1205–1227. 10.1037/a0039650 26390265

[B146] Kaza N. (2010). Understanding the spectrum of residential energy consumption: A quantile regression approach. Energy Policy 38, 6574–6585. 10.1016/j.enpol.2010.06.028

[B147] Keizer M. Sargisson R. J. van Zomeren M. Steg L. (2019). When personal norms predict the acceptability of push and pull car-reduction policies: Testing the ABC model and low-cost hypothesis. Transport. Res. Part F 64, 413–423. 10.1016/j.trf.2019.06.005

[B148] Komatsu H. Nishio K.-i. (2015). An experimental study on motivational change for electricity conservation by normative messages. Appl. Energy 158, 35–43. 10.1016/j.apenergy.2015.08.029

[B149] Korcaj L. Hahnel U. J. Spada H. (2015). Intentions to adopt photovoltaic systems depend on homeowners' expected personal gains and behavior of peers. Renew. Energy 75, 407–415. 10.1016/j.renene.2014.10.007

[B150] Kormos C. Axsen J. Long Z. Goldberg S. (2019). Latent demand for zero-emissions vehicles in Canada (Part 2): Insights from a stated choice experiment. Transport. Res. Part D 67, 685–702. 10.1016/j.trd.2018.10.010

[B151] Kormos C. Gifford R. (2014). The validity of self-report measures of proenvironmental behavior: A meta-analytic review. J. Environ. Psychol. 40, 359–371. 10.1016/j.jenvp.2014.09.003

[B152] Krause R. M. Carley S. R. Lane B. W. Graham J. D. (2013). Perception and reality: Public knowledge of plug-in electric vehicles in 21 U.S. cities. Energy Policy 63, 433–440. 10.1016/j.enpol.2013.09.018

[B153] Labandeira X. Labeaga J. M. Linares P. López-Otero X. (2020). The impacts of energy efficiency policies: Meta-analysis. Energy Policy 147, 111790. 10.1016/j.enpol.2020.111790

[B154] Labandeira X. Labeaga J. M. López-Otero X. (2017). A meta-analysis on the price elasticity of energy demand. Energy Policy 102, 549–568. 10.1016/j.enpol.2017.01.00235648401

[B155] Lapinski M. K. Zhuang J. Koh H. Shi J. (2017). Descriptive norms and involvement in health and environmental behaviors. Communic. Res. 44, 367–387. 10.1177/0093650215605153

[B156] Lavergne K. J. Sharp E. C. Pelletier L. G. Holtby A. (2010). The role of perceived government style in the facilitation of self-determined and non self-determined motivation for pro-environmental behavior. J. Environ. Psychol. 30, 169–177. 10.1016/j.jenvp.2009.11.002

[B157] Legault L. Bird S. Powers S. E. Sherman A. Schay A. Hou D. . (2020). Impact of a motivational intervention and interactive feedback on electricity and water consumption: A smart housing field experiment. Environ. Behav. 52, 666–692. 10.1177/0013916518811433

[B158] Leoniak K. J. Cwalina W. (2019). The role of normative prompts and norm support cues in promoting light-switching behavior: A field study. J. Environ. Psychol. 64, 1–11. 10.1016/j.jenvp.2019.04.014

[B159] Levitt S. D. List J. A. (2007). What do laboratory experiments measuring social preferences tell us about the real world? J. Econ. Perspect. 21, 153–174. 10.1257/jep.21.2.153

[B160] List J. A. Metcalfe R. D. Price M. K. Rundhammer F. (2017). Harnessing Policy Complementarities to Conserve Energy: Evidence From a Natural Field Experiment. Working paper. 10.3386/w23355

[B161] Liu Y. Q. Verissimo D. Farhidi F. (2016). Using social norm to promote energy conservation in a public building. Energy Build. 133, 32–36. 10.1016/j.enbuild.2016.09.041

[B162] Locke E. A. Latham G. P. (2002). Building a practically useful theory of goal setting and task motivation: A 35-year odyssey. Am. Psychol. 57, 705–717. 10.1037/0003-066X.57.9.705 12237980

[B163] Lokhorst A. M. Staats H. van Iterson J. (2015). Energy saving in office buildings: Are feedback and commitment-making useful instruments to trigger change? Hum. Ecol. 43, 759–768. 10.1007/s10745-015-9783-8 26543301 PMC4624829

[B164] Lokhorst A. M. Werner C. Staats H. van Dijk E. Gale J. L. (2013). Commitment and behavior change: A meta-analysis and critical review of commitment-making strategies in environmental research. Environ. Behav. 45, 3–34. 10.1177/0013916511411477

[B165] Loock C.-M. Landwehr J. R. Staake T. Fleisch E. Pentland A. S. (2012). The influence of reference frame and population density on the effectiveness of social normative feedback on electricity consumption, in: Proceedings of the International Conference on Information Systems (ICIS) (Orlando, FL).

[B166] Loock C.-M. Staake T. Thiesse F. (2013). Motivating energy-efficient behavior with green IS: An investigation of goal setting and the role of defaults. MIS Quart. 37, 1313–1332. 10.25300/MISQ/2013/37.4.15

[B167] Löschel A. Rodemeier M. Werthschulte M. (2020). When Nudges Fail to Scale: Field Experimental Evidence From Goal Setting on Mobile Phones. Working paper. 10.2139/ssrn.3676090

[B168] Lundgren B. Schultzberg M. (2019). Application of the economic theory of self-control to model energy conservation behavioral change in households. Energy 183, 536–546. 10.1016/j.energy.2019.05.217

[B169] Lundheim S. H. Vesely S. Nayum A. Klöckner C. A. (2021). From vague interest to strong intentions to install solar panels on private homes in the North – An analysis of psychological drivers. Renew. Energy 165, 455–463. 10.1016/j.renene.2020.11.034

[B170] Mack B. Tampe-Mai K. Kouros J. Roth F. Taube O. Diesch E. (2019). Bridging the electricity saving intention-behavior gap: A German field experiment with a smart meter website. Energy Res. Soc. Sci. 53, 34–46. 10.1016/j.erss.2019.01.024

[B171] MacKinnon D. P. (2011). Integrating mediators and moderators in research design. Res. Soc. Work Pract. 21, 675–681. 10.1177/1049731511414148 22675239 PMC3366634

[B172] Mahmoodi J. Prasanna A. Hille S. Patel M. K. Brosch T. (2018). Combining “carrot and stick” to incentivize sustainability in households. Energy Policy 123, 31–40. 10.1016/j.enpol.2018.08.037

[B173] Maki A. Carrico A. R. Raimi K. T. Truelove H. B. Araujo B. Yeung K. L. (2019). Meta-analysis of pro-environmental behavior spillover. Nat. Sust. 2, 307–315. 10.1038/s41893-019-0263-9

[B174] Maniadis Z. Tufano F. List J. A. (2014). One swallow doesn't make a summer: New evidence on anchoring effects. Am. Econ. Rev. 104, 277–290. 10.1257/aer.104.1.277

[B175] Martin S. Rivers N. (2018). Information provision, market incentives, and household electricity consumption: Evidence from a larg*e*-scale field deployment. J. Assoc. Environ. Resour. Econ. 5, 207–231. 10.1086/694036

[B176] Masson T. Fritsche I. (2014). Adherence to climate change-related ingroup norms: Do dimensions of group identification matter? Eur. J. Soc. Psychol. 44, 455–465. 10.1002/ejsp.2036

[B177] Matthies E. Klöckner C. A. Preißner C. L. (2006). Applying a modified moral decision making model to change habitual car use: How can commitment be effective? Appl. Psychol. 55, 91–106. 10.1111/j.1464-0597.2006.00237.x

[B178] McCoy D. Lyons S. (2017). Unintended outcomes of electricity smart-metering: Trading-off consumption and investment behaviour. Energy Effic. 10, 299–318. 10.1007/s12053-016-9452-9

[B179] McKenna R. Hernando D. A. ben Brahim T. Bolwig S. Cohen J. J. Reichl J. (2021). Analyzing the energy system impacts of price-induced demand-side-flexibility with empirical data. J. Clean. Prod. 279, 123354. 10.1016/j.jclepro.2020.123354

[B180] Meier H. Rehdanz K. (2010). Determinants of residential space heating expenditures in Great Britain. Energy Econ. 32, 949–959. 10.1016/j.eneco.2009.11.008

[B181] Mertens S. N. Schultz P. W. (2021). Referent group specificity: Optimizing normative feedback to increase residential recycling. J. Environ. Psychol. 73, 101541. 10.1016/j.jenvp.2020.101541

[B182] Mi L. Ding C. Yang J. Yu X. Cong J. Zhu H. . (2019). Using goal and contrast feedback to motivate Chinese urban families to save electricity actively – a randomized controlled field trial. J. Clean. Prod. 226, 443–453. 10.1016/j.jclepro.2019.04.068

[B183] Mi L. Y. Qiao L. J. Du S. S. Xu T. Gan X. L. Wang W. S. . (2020a). Evaluating the effect of eight customized information strategies on urban households' electricity saving: A field experiment in China. Sust. Cities Soc. 62, 102344. 10.1016/j.scs.2020.102344

[B184] Mi L. Y. Qiao L. J. Gan X. L. Xu T. Lv T. Qiao Y. N. . (2020b). Assessing the effect of non-financial information intervention on promoting group-level energy savings. Sci. Total Environ. 720, 137533. 10.1016/j.scitotenv.2020.137533 32135279

[B185] Miller C. K. (2019). Adaptive intervention designs to promote behavioral change in adults: What is the evidence? Curr. Diab. Rep. 19, 7–16. 10.1007/s11892-019-1127-4 30684109

[B186] Mills B. Schleich J. (2012). Residential energy-efficient technology adoption, energy conservation, knowledge, and attitudes: An analysis of European countries. Energy Policy 49, 616–628. 10.1016/j.enpol.2012.07.008

[B187] Mitchell G. (2012). Revisiting truth or triviality: The external validity of research in the psychological laboratory. Perspect. Psychol. Sci. 7, 109–117. 10.1177/1745691611432343 26168439

[B188] Moons I. De Pelsmacker P. (2012). Emotions as determinants of electric car usage intention. J. Market. Manag. 28, 195–237. 10.1080/0267257X.2012.659007

[B189] Moons I. De Pelsmacker P. (2015). An extended decomposed theory of planned behaviour to predict the usage intention of the electric car: A multi-group comparison. Sustainability 7, 6212–6245. 10.3390/su7056212

[B190] Moshiri S. (2015). The effects of the energy price reform on households consumption in Iran. Energy Policy 79, 177–188. 10.1016/j.enpol.2015.01.01229445697

[B191] Münzel C. Plötz P. Sprei F. Gnann T. (2019). How large is the effect of financial incentives on electric vehicle sales? – a global review and European analysis. Energy Econ. 84, 104493. 10.1016/j.eneco.2019.104493

[B192] Murakami K. Shimada H. Ushifusa Y. Ida T. (2022). Heterogeneous treatment effects of nudge and rebate: Causal machine learning in a field experiment on electricity conservation. Int. Econ. Rev. 10.1111/iere.12589 [Epub ahead of print].

[B193] Myers E. Souza M. (2020). Social comparison nudges without monetary incentives: Evidence from home energy reports. J. Environ. Econ. Manag. 101, 102315. 10.1016/j.jeem.2020.102315

[B194] Nahum-Shani I. Smith S. N. Spring B. J. Collins L. M. Witkiewitz K. Tewari A. . (2018). Just-in-Time Adaptive Interventions (JITAIs) in mobile health: Key components and design principles for ongoing health behavior support. Ann. Behav. Med. 52, 446–462. 10.1007/s12160-016-9830-8 27663578 PMC5364076

[B195] Nemati M. Penn J. (2020). The impact of information-based interventions on conservation behavior: A meta-analysis. Resour. Energy Econ. 62, 101201. 10.1016/j.reseneeco.2020.101201

[B196] Nesbakken R. (1999). Price sensitivity of residential energy consumption in Norway. Energy Econ. 21, 493–515. 10.1016/S0140-9883(99)00022-5

[B197] Neubert M. J. (1998). The value of feedback and goal setting over goal setting alone and potential moderators of this effect: A meta-analysis. Human Perform. 11, 321–335. 10.1207/s15327043hup1104_2

[B198] Neumann R. Mehlkop G. (2020). Framing electricity plan choices to enhance green energy usage: A choice experiment with panel data from Germany. Energy Res. Soc. Sci. 70, 101741. 10.1016/j.erss.2020.101741

[B199] Nisa C. F. Bélanger J. J. Schumpe B. M. Faller D. G. (2019). Meta-analysis of randomised controlled trials testing behavioural interventions to promote household action on climate change. Nat. Commun. 10, 4545. 10.1038/s41467-019-12457-2 31586060 PMC6778105

[B200] Noppers E. Keizer K. Milovanovic M. Steg L. (2019). The role of adoption norms and perceived product attributes in the adoption of Dutch electric vehicles and smart energy systems. Energy Res. Soc. Sci. 57, 101237. 10.1016/j.erss.2019.101237

[B201] Ojima K. Akashi Y. Lim J. Yoshimoto N. Chen J. (2019). Effect of energy information provision on occupant's behavior and energy consumption in public spaces. IOP Conf. Series Earth Environ. Sci. 294, 012080. 10.1088/1755-1315/294/1/012080

[B202] Ornaghi C. Costanza E. Kittley-Davies J. Bourikas L. Aragon V. James P. A. B. (2018). The effect of behavioural interventions on energy conservation in naturally ventilated offices. Energy Econ. 74, 582–591. 10.1016/j.eneco.2018.07.008

[B203] Osbaldiston R. Schott J. P. (2012). Environmental sustainability and behavioral science: Meta-analysis of proenvironmental behavior experiments. Environ. Behav. 44, 257–299. 10.1177/0013916511402673

[B204] Pallak M. S. Cummings W. (1976). Commitment and voluntary energy conservation. Pers. Soc. Psychol. Bull. 2, 27–30. 10.1177/014616727600200105

[B205] Palmer K. Tate J. E. Wadud Z. Nellthorp J. (2018). Total cost of ownership and market share for hybrid and electric vehicles in the UK, US and Japan. Appl. Energy 209, 108–119. 10.1016/j.apenergy.2017.10.089

[B206] Passafaro P. Livi S. Kosic A. (2019). Local norms and the theory of planned behavior: Understanding the effects of spatial proximity on recycling intentions and self-reported behavior. Front. Psychol. 10, 744. 10.3389/fpsyg.2019.00744 30984093 PMC6450212

[B207] Pellerano J. A. Price M. K. Puller S. L. Sánchez G. E. (2017). Do extrinsic incentives undermine social norms? Evidence from a field experiment in energy conservation. Environ. Resour. Econ. 67, 413–428. 10.1007/s10640-016-0094-3

[B208] Prest B. C. (2020). Peaking interest: How awareness drives the effectiveness of time-of-use electricity pricing. J. Assoc. Environ. Resour. Econ. 7, 103–143. 10.1086/705798

[B209] Rehdanz K. (2007). Determinants of residential space heating expenditures in Germany. Energy Econ. 29, 167–182. 10.1016/j.eneco.2006.04.002

[B210] Reiss P. C. White M. W. (2005). Household electricity demand, revisited. Rev. Econ. Stud. 72, 853–883. 10.1111/0034-6527.00354

[B211] Rezvani Z. Jansson J. Bengtsson M. (2018). Consumer motivations for sustainable consumption: The interaction of gain, normative and hedonic motivations on electric vehicle adoption. Business Strat. Environ. 27, 1272–1283. 10.1002/bse.2074

[B212] Rhodes E. Axsen J. Jaccard M. (2017). Exploring citizen support for different types of climate policy. Ecol. Econ. 137, 56–69. 10.1016/j.ecolecon.2017.02.027

[B213] Rodemeier M. Löschel A. (2020). The Welfare Effects of Persuasion and Taxation: Theory and Evidence From the Field. Working paper. 10.2139/ssrn.3594011

[B214] Royal A. Rustamov G. (2018). Do small pecuniary incentives motivate residential peak energy reductions? Experimental evidence. Appl. Econ. 50, 6193–6202. 10.1080/00036846.2018.1489508

[B215] Ščasný M. Zvěrinová I. Czajkowski M. (2018). Electric, plug-in hybrid, hybrid, or conventional? Polish consumers' preferences for electric vehicles. Energy Effic. 11, 2181–2201. 10.1007/s12053-018-9754-1

[B216] Schmitz H. Madlener R. (2020). Heterogeneity in price responsiveness for residential space heating in Germany. Empir. Econ. 59, 2255–2281. 10.1007/s00181-019-01760-y

[B217] Schulte I. Heindl P. (2017). Price and income elasticities of residential energy demand in Germany. Energy Policy 102, 512–528. 10.1016/j.enpol.2016.12.055

[B218] Schultz P. W. Estrada M. Schmitt J. Sokoloski R. Silva-Send N. (2015). Using in-home displays to provide smart meter feedback about household electricity consumption: A randomized control trial comparing kilowatts, cost, and social norms. Energy 90, 351–358. 10.1016/j.energy.2015.06.130

[B219] Schultz P. W. Messina A. Tronu G. Limas E. F. Gupta R. Estrada M. (2016). Personalized normative feedback and the moderating role of personal norms: a field experiment to reduce residential water consumption. Environ. Behav. 48, 686–710. 10.1177/0013916514553835

[B220] Schultz P. W. Nolan J. M. Cialdini R. B. Goldstein N. J. Griskevicius V. (2007). The constructive, destructive, and reconstructive power of social norms. Psychol. Sci. 18, 429–434. 10.1111/j.1467-9280.2007.01917.x 17576283

[B221] Schultz W. P. Khazian A. M. Zaleski A. C. (2008). Using normative social influence to promote conservation among hotel guests. Soc. Infl. 3, 4–23. 10.1080/15534510701755614

[B222] Schwartz D. Bruine de Bruin W. Fischhoff B. Lave L. (2015). Advertising energy saving programs: The potential environmental cost of emphasizing monetary savings. J. Exp. Psychol. Appl. 21, 158–166. 10.1037/xap0000042 25581089

[B223] Sexton R. J. Sexton T. A. Wann J. J.-W. Kling C. L. (1989). The conservation and welfare effects of information in a time-of-day pricing experiment. Land Econ. 65, 272–279. 10.2307/3146671

[B224] Shen M. Lu Y. Law Y. E. (2019). The effect of goal setting strategy and residents' goal commitment on household electricity consumption in Singapore, in: Proceedings of 11th International Conference on Applied Energy (Västerås, Sweden).

[B225] Shen M. Young R. Cui Q. (2016). The normative feedback approach for energy conservation behavior in the military community. Energy Policy 98, 19–32. 10.1016/j.enpol.2016.08.014

[B226] Silva S. Soares I. Pinho C. (2018). Electricity residential demand elasticities: Urban versus rural areas in Portugal. Energy 144, 627–632. 10.1016/j.energy.2017.12.070

[B227] Sovacool B. K. Abrahamse W. Zhang W. Ren J. (2019). Pleasure or profit? Surveying the purchasing intentions of potential electric vehicle adopters in China. Transport. Res. Part A 124, 69–81. 10.1016/j.tra.2019.03.002

[B228] Sovacool B. K. Kester J. Noel L. de Rubens G. Z. (2018). The demographics of decarbonizing transport: The influence of gender, education, occupation, age, and household size on electric mobility preferences in the Nordic region. Glob. Environ. Change 52, 86–100. 10.1016/j.gloenvcha.2018.06.008

[B229] Stanley T. D. Carter E. C. Doucouliagos H. (2018). What meta-analyses reveal about the replicability of psychological research. Psychol. Bull. 144, 1325–1346. 10.1037/bul0000169 30321017

[B230] Steinhorst J. Klöckner C. A. (2018). Effects of monetary versus environmental information framing: Implications for long-term pro-environmental behavior and intrinsic motivation. Environ. Behav. 50, 997–1031. 10.1177/0013916517725371

[B231] Steinhorst J. Klöckner C. A. Matthies E. (2015). Saving electricity – FOR the money or the environment? Risks of limiting pro-environmental spillover when using monetary framing. J. Environ. Psychol. 43, 125–135. 10.1016/j.jenvp.2015.05.012

[B232] Steinhorst J. Matthies E. (2016). Monetary or environmental appeals for saving electricity? – potentials for spillover on low carbon policy acceptability. Energy Policy 93, 335–344. 10.1016/j.enpol.2016.03.020

[B233] Stern P. C. Janda K. B. Brown M. A. Steg L. Vine E. L. Lutzenhiser L. (2016). Opportunities and insights for reducing fossil fuel consumption by households and organizations. Nat. Energy 1, 16043. 10.1038/nenergy.2016.43

[B234] Stojanovski O. Leslie G. W. Wolak F. A. Wong J. E. H. Thurber M. C. (2020). Increasing the energy cognizance of electricity consumers in Mexico: Results from a field experiment. J. Environ. Econ. Manag. 102, 102323. 10.1016/j.jeem.2020.102323

[B235] Sudarshan A. (2017). Nudges in the marketplace: The response of household electricity consumption to information and monetary incentives. J. Econ. Behav. Organiz. 134, 320–335. 10.1016/j.jebo.2016.12.015

[B236] Sussman R. Gifford R. (2012). Please turn off the lights: The effectiveness of visual prompts. Appl. Ergon. 43, 596–603. 10.1016/j.apergo.2011.09.008 21963251

[B237] Suter J. F. Shammin M. R. (2013). Returns to residential energy efficiency and conservation measures: A field experiment. Energy Policy 59, 551–561. 10.1016/j.enpol.2013.04.003

[B238] Terrier L. Marfaing B. (2015). Using social norms and commitment to promote pro-environmental behavior among hotel guests. J. Environ. Psychol. 44, 10–15. 10.1016/j.jenvp.2015.09.001

[B239] Terry D. J. Hogg M. A. White K. M. (1999). The theory of planned behaviour: Self-identity, social identity and group norms. Br. J. Soc. Psychol. 38, 225–244. 10.1348/014466699164149 10520477

[B240] Tiefenbeck V. Staake T. Roth K. Sachs O. (2013). For better or for worse? Empirical evidence of moral licensing in a behavioral energy conservation campaign. Energy Policy 57, 160–171. 10.1016/j.enpol.2013.01.021

[B241] Tobler C. Visschers V. H. M. Siegrist M. (2012). Addressing climate change: Determinants of consumers' willingness to act and to support policy measures. J. Environ. Psychol. 32, 197–207. 10.1016/j.jenvp.2012.02.001

[B242] Todd-Blick A. Spurlock C. A. Jin L. Cappers P. Borgeson S. Fredman D. . (2020). Winners are not keepers: Characterizing household engagement, gains, and energy patterns in demand response using machine learning in the United States. Energy Res. Soc. Sci. 70, 101595. 10.1016/j.erss.2020.101595

[B243] Toner K. Gan M. Leary M. R. (2014). The impact of individual and group feedback on environmental intentions and self-beliefs. Environ. Behav. 46, 24–45. 10.1177/0013916512451902

[B244] van den Broek K. Bolderdijk J. W. Steg L. (2017). Individual differences in values determine the relative persuasiveness of biospheric, economic and combined appeals. J. Environ. Psychol. 53, 145–156. 10.1016/j.jenvp.2017.07.009

[B245] van der Werff E. Taufik D. Venhoeven L. (2019). Pull the plug: How private commitment strategies can strengthen personal norms and promote energy-saving in the Netherlands. Energy Res. Soc. Sci. 54, 26–33. 10.1016/j.erss.2019.03.002

[B246] Vesely S. Klöckner C. A. (2018). How anonymity and norms influence costly support for environmental causes. J. Environ. Psychol. 58, 27–30. 10.1016/j.jenvp.2018.07.013

[B247] Vesely S. Klöckner C. A. (2020). Social desirability in environmental psychology research: Three meta-analyses. Front. Psychol. 11, 1395. 10.3389/fpsyg.2020.01395 32793022 PMC7393925

[B248] Vesely S. Klöckner C. A. Carrus G. Chokrai P. Fritsche I. Masson T. . (2022). Donations to renewable energy projects: The role of social norms and donor anonymity. Ecol. Econ. 193, 107277. 10.1016/j.ecolecon.2021.107277

[B249] Wan C. Shen G. Q. Choi S. (2017). Experiential and instrumental attitudes: Interaction effect of attitude and subjective norm on recycling intention. J. Environ. Psychol. 50, 69–79. 10.1016/j.jenvp.2017.02.006

[B250] West J. Fairlie R. W. Pratt B. Rose L. (2021). Automated enforcement of irrigation regulations and social pressure for water conservation. J. Assoc. Environ. Resour. Economists 8, 1179–1207. 10.1086/715472

[B251] White K. Simpson B. (2013). When do (and don't) normative appeals influence sustainable consumer behaviors? J. Mark. 77, 78–95. 10.1509/jm.11.0278

[B252] White K. M. Smith J. R. Terry D. J. Greenslade J. H. McKimmie B. M. (2009). Social influence in the theory of planned behaviour: The role of descriptive, injunctive, and in-group norms. Br. J. Soc. Psychol. 48, 135–158. 10.1348/014466608X295207 18435863

[B253] White L. V. Sintov N. D. (2020). Health and financial impacts of demand-side response measures differ across sociodemographic groups. Nat. Energy 5, 50–60. 10.1038/s41560-019-0507-y

[B254] Wichman C. J. Ferraro P. J. (2017). A cautionary tale on using panel data estimators to measure program impacts. Econ. Lett. 151, 82–90. 10.1016/j.econlet.2016.11.029

[B255] Wichman C. J. Taylor L. O. von Haefen R. H. (2016). Conservation policies: Who responds to price and who responds to prescription? J. Environ. Econ. Manag. 79, 114–134. 10.1016/j.jeem.2016.07.001

[B256] Wolske K. S. (2020). More alike than different: Profiles of high-income and low-income rooftop solar adopters in the United States. Energy Res. Soc. Sci. 63, 101399. 10.1016/j.erss.2019.101399

[B257] Wolske K. S. Stern P. C. Dietz T. (2017). Explaining interest in adopting residential solar photovoltaic systems in the United States: Toward an integration of behavioral theories. Energy Res. Soc. Sci. 25, 134–151. 10.1016/j.erss.2016.12.023

[B258] Wong-Parodi G. Krishnamurti T. Gluck J. Agarwal Y. (2019). Encouraging energy conservation at work: A field study testing social norm feedback and awareness of monitoring. Energy Policy 130, 197–205. 10.1016/j.enpol.2019.03.028

[B259] Woo C. K. Li R. Shiu A. Horowitz I. (2013). Residential winter kW h responsiveness under optional time-varying pricing in British Columbia. Appl. Energy 108, 288–297. 10.1016/j.apenergy.2013.03.042

[B260] Xu X. Arpan L. M. Chen C.-f. (2015). The moderating role of individual differences in responses to benefit and temporal framing of messages promoting residential energy saving. J. Environ. Psychol. 44, 95–108. 10.1016/j.jenvp.2015.09.004

[B261] Yang S. Zhao D. (2015). Do subsidies work better in low-income than in high-income families? Survey on domestic energy-efficient and renewable energy equipment purchase in China. J. Clean. Prod. 108, 841–851. 10.1016/j.jclepro.2015.07.022

[B262] Yeomans M. Herberich D. (2014). An experimental test of the effect of negative social norms on energy-efficient investments. J. Econ. Behav. Organiz. 108, 187–197. 10.1016/j.jebo.2014.09.010

[B263] Zhang F. (2015). Energy price reform and household welfare: The case of Turkey. Energy J. 36, 71–96. 10.5547/01956574.36.2.4

[B264] Zhang Y. Qian Z. Sprei F. Li B. (2016). The impact of car specifications, prices and incentives for battery electric vehicles in Norway: Choices of heterogeneous consumers. Transport. Res. Part C 69, 386–401. 10.1016/j.trc.2016.06.014

[B265] Zhu X. Li L. Zhou K. Zhang X. Yang S. (2018). A meta-analysis on the price elasticity and income elasticity of residential electricity demand. J. Clean. Prod. 201, 169–177. 10.1016/j.jclepro.2018.08.027

