# Supplementary material for: Norms, prices, and commitment: A comprehensive overview of field experiments in the energy domain and treatment effect moderators
Source: Front Psychol. 2022 Nov 8;13:967318. doi: 10.3389/fpsyg.2022.967318 (PMC9680531; doi:10.3389/fpsyg.2022.967318)
Supplement: Supplementary file 1 [file Table_1.docx]

Supplementary Table 1: Overview of former research – incentives and costs

| Source | Main methodology used | Main target behavior(s) | Type of incentive or cost | Effect of incentive or cost confirmed? |
| --- | --- | --- | --- | --- |
| Buckley (2020) | Meta-analysis of field experimental studies | Energy conservation | Monetary information (information on electricity consumption costs) | Small increase (sic!) in electricity consumption (meta-regression results, full model specification) |
|  |  |  | Various pricing strategies | No effect (meta-regression results, full model specification) |
| Delmas et al. (2013) | Meta-analysis of field experimental studies | Energy conservation | Monetary information (information on potential savings from energy conservation) | No effect (meta-regression results, full model specification) |
|  |  |  | Various incentives, including rebates, cash rewards, and tiered electricity pricing | Small increase (sic!) in energy consumption (meta-regression results, full model specification) |
| Faruqui & Sergici (2010) | Meta-analysis of field experimental studies | Energy conservation | Time-of-use electricity pricing | Small decrease in energy consumption |
|  |  |  | Critical-peak electricity pricing | Large drop in electricity demand during peak hours |
| Allcott & Greenstone (2017) | Field experiment | Takeup of home energy audits | Subsidized audit price | Large positive effect (result for a $100 subsidy) |
|  |  |  | Cash reward for participating in the energy audit | No effect |
|  |  |  | Monetary information (information on economic benefits of energy efficiency improvements and on available financing options) | No effects (results for the following treatments: “info: financial”, “financing: credit”, “financing: incentives”, and “prime: financial”) |
|  |  | Investment in home energy efficiency improvements | Subsidized audit price | No effect |
|  |  |  | Cash reward for participating in an energy audit | No effect |
|  |  |  | Monetary information (information on economic benefits of energy efficiency improvements and on available financing options) | No effects (results for the following treatments: “info: financial”, “financing: credit”, “financing: incentives”, and “prime: financial”) |
| Allcott & Sweeney (2017) | Field experiment | Purchasing energy-efficient water heaters | Monetary information (information on financial savings over time from buying an efficient water heater) | No effect |
|  |  |  | Rebate | No effect (result for a $25 rebate)  Large increase in the probability to buy an efficient model (result for a $100 rebate) |
| Allcott & Taubinsky (2015) | Field experiment | Purchasing energy-efficient light bulbs | Monetary information (information on total user costs, including energy usage costs, for different types of light bulbs) | No effect |
|  |  |  | Rebate | Large increase in demand for energy-efficient light bulbs |
| Azarova et al. (2020) | Field experiment | Energy conservation during a 15-minute peak period | Reward in the form of seven days of free electricity if 50 percent consumption reduction is achieved | No effect (“monetary incentive” treatment effect estimated in the full model with date fixed effects; rebound effects not considered here) |
| Bollinger et al. (2020b) | Field experiment | Adoption of residential solar panels | Monetary information (information on economic and also on other non-financial benefits) | Large increase in installations relative to control (longer-term post-campaign effects not considered here) |
| Bollinger & Hartmann (2020) | Field experiment | Energy conservation during peak-demand hours | Time-of-use pricing, variable peak pricing | Moderate to large decrease in energy consumption during critical peak periods (depending on the tariff used); no effect during non-critical peak and off-peak periods (treatment effect regression results for the “Portal” condition relative to control) |
|  |  |  | Variable peak pricing with critical pricing | Moderate decrease in energy consumption during critical peak periods; no effect during non-critical peak and off-peak periods (treatment effect regression results for the “Portal” condition relative to control) |
| Burkhardt et al. (2019) | Field experiment | Energy conservation during peak events | Critical peak pricing | Large decrease in electricity consumption during peak events |
| d’Adda et al. (2020) | Field experiment | Purchasing energy-efficient refrigerators | Monetary information (information on energy usage costs for different available products) | Small drop (sic!) in demand for energy-efficient products (result from ordered probit regression aggregating across monetary information treatments) |
| Di Cosmo et al. (2014) | Field experiment | Energy conservation during peak-demand hours | Time-of-use electricity pricing | Decrease in consumption during peak-demand periods (for four out of five tariffs used, no effect in the fifth case; results for bi-monthly billing; off-peak effects not considered here) |
| Faruqui et al. (2013) | Field experiment | Energy conservation during peak events | Critical peak pricing | Large drop in electricity consumption during peak events |
|  |  |  | Peak time rebate | Large drop in electricity consumption during peak events |
| Faruqui et al. (2014) | Field experiment | Energy conservation during peak periods | Critical peak pricing | Large decrease in electricity consumption during peak periods for residential customers; small to moderate decrease for commercial and industrial customers |
|  |  |  | Peak time rebate | Moderate to large decrease in electricity consumption during peak periods for residential customers; small decrease for commercial and industrial customers |
|  |  |  | Time-of-use electricity pricing | Small decrease in electricity consumption during peak periods for residential customers; no effect for commercial and industrial customers |
| Fenrick et al. (2014) | Field experiment | Energy conservation during peak events | Critical peak pricing (opt-in design) | Large decrease in electricity consumption during peak events |
|  |  |  | Critical peak pricing (opt-out design) | Moderate decrease in electricity consumption during peak events |
| Figueroa et al. (2019) | Field experiment | Purchasing energy-efficient light bulbs | Rebate | Large increase in demand for energy-efficient light bulbs (long-term effects not considered here) |
| Fowlie et al. (2020) | Field experiment | Energy conservation during peak events | Critical peak pricing (opt-in design) | Moderate decrease in electricity consumption during peak events (intent-to-treat analysis) |
|  |  |  | Critical peak pricing (opt-out design) | Large decrease in electricity consumption during peak events (intent-to-treat analysis) |
|  |  | Energy conservation | Time-of-use electricity pricing | Small to moderate decrease in electricity consumption |
| Gillan (2018) | Field experiment | Energy conservation during peak events | Critical peak pricing | Moderate to large drop in electricity consumption during peak events (depending on the use of automation technology; intent-to-treat estimates for the first 90 days of the program) |
|  |  | Adoption of smart energy technology (smart thermostats and smart plugs) | Rebate | Large increase in demand for the technologies |
| Gillingham & Bollinger (in press) | Field experiment | Adoption of residential solar panels | Group pricing discount (an installation’s price is a function of the total number of contracts signed as part of the campaign) | No effect (comparison of the “no group pricing” campaigns and the Round 5 “classic Solarize” campaigns) |
| Grimm et al. (2020) | Field experiment | Purchasing solar kits | Extended payment period (i.e., possibility to pay for a product later rather than at the time of purchase) | No effect of longer payment periods in five out of six cases, moderate increase in demand in one case |
| Harding & Lamarche (2016) | Field experiment | Energy conservation during peak-demand hours | Time-of-use electricity pricing | No effect (result for the “Portal” condition relative to control) |
| Harding et al. (2020) | Field experiment | Energy conservation during peak-demand hours | Time-of-use electricity pricing | Small decrease in energy consumption (result for the “Portal” condition relative to control) |
| Harold et al. (2018) | Field experiment | Energy conservation | Variable tariff | No effect (variable tariff with in-home display compared to standard tariff with in-home display) |
| Holladay et al. (2019) | Field experiment | Takeup of home energy audits | Reward for participating in the energy audit (gift card) | No effect (result for a $20 reward)  Large, marginally significant increase in audit takeup (result for a $50 reward) |
|  |  | Investment in home energy efficiency improvements | Reward for participating in an energy audit (gift card) | No effect |
| Ida et al. (2016) | Field experiment | Energy conservation during peak events | Critical peak pricing | Small decrease in electricity consumption during peak events |
| Ito et al. (2018) | Field experiment | Energy conservation during peak-demand hours | Critical-peak electricity pricing (increased electricity price during peak hours) | Large decrease in electricity consumption |
| List et al. (2017) | Field experiment | Energy conservation | Offer of rewards (e.g., gift cards) for reductions in energy usage | Small, marginally significant decrease in energy consumption (intent-to-treat analysis) |
| Mi et al. (2020a) | Field experiment | Energy conservation | Monetary information (money spent on electricity) | Large, marginally significant decrease in energy consumption (result for the “cost-benefit feedback” condition) |
| Murakami et al. (2020) | Field experiment | Energy conservation during peak-demand hours | Rebate for reduced electricity use in peak-demand hours | Small decrease in electricity consumption during peak-demand periods |
| Pellerano et al. (2017) | Field experiment | Energy conservation | Monetary information (information on the participant’s electricity tariff and on potential savings from energy conservation) | No effect |
| Prest (2020) | Field experiment | Energy conservation during peak-demand hours | Time-of-use electricity pricing | Moderate decrease in electricity consumption during peak-demand periods |
| Rodemeier & Löschel (2020) | Field experiment | Purchasing energy-efficient light bulbs | Monetary information (information on energy usage monetary savings for different types of light bulbs) | Less detailed information treatment: No effect (results from regressions on pooled treatments)  More detailed information treatment: Large drop (sic!) in demand for the most efficient light bulb, large increase in demand for the second most efficient light bulb, and large drop in demand for the least efficient light bulb (results from regressions on pooled treatments) |
|  |  |  | Discount | Large increase in demand for the most efficient light bulb, no effect of discounts on other types of light bulbs (results from regressions on pooled treatments) |
| Royal & Rustamov (2018) | Field experiment | Energy conservation during peak-demand events | Critical-peak electricity pricing | Large decrease in electricity consumption during peak-demand events |
| Stojanovski et al. (2020) | Field experiment | Energy conservation | Monetary information (information on the participant’s electricity tariff and on potential monetary savings from energy conservation) | Small, marginally significant decrease in energy consumption (intent-to-treat analysis) |
| Sudarshan (2017) | Field experiment | Energy conservation | Rewards for below-average and penalties for above-average electricity consumption | Moderate increase (sic!) in consumption (incremental effect of incentives compared to the “information only” treatment; compared to control, which is a less appropriate comparison here, there was no effect) |
| Suter & Shammin (2013) | Field experiment | Energy conservation | Rewards for reducing gas consumption | Large decrease in gas consumption |
| Todd-Blick et al. (2020) | Field experiment | Energy conservation during critical peak hours | Time-of-use electricity pricing | Decrease in electricity consumption during critical peak hours (treatment-on-the-treated estimate) |
| Yoeli et al. (2013) | Field experiment | Allowing the utility company to remotely switch off one’s air conditioning during peak-demand events | Financial reward | No effect |
| Wang et al. (2020) | Field experiment | Energy conservation during peak events | Critical peak electricity pricing | Moderate decrease in electricity consumption during peak events (intent-to-treat analysis) |
| White & Sintov (2020) | Field experiment | Energy conservation during peak-demand hours | Time-of-use electricity pricing | Decrease in electricity consumption during peak-demand hours |
| Wolak (2011) | Field experiment | Energy conservation | Critical peak pricing, and critical peak pricing plus rebate | Moderate to large decrease in energy consumption (depending on the sample) |

References not cited in the main text:

Allcott, H., and Greenstone, M. (2017). Measuring the Welfare Effects of Residential Energy Efficiency Programs With Self-Selection Into Program Participation. Working paper. doi: 10.3386/w23386

d’Adda, G., Gao, Y., and Tavoni, M. (2020). Making Energy Costs Salient Can Lead to Low-Efficiency Purchases. Working paper (E2e Project Working Paper Series).

Di Cosmo, V., Lyons, S., and Nolan, A. (2014). Estimating the impact of time-of-use pricing on Irish electricity demand. Energy J. 35, 117–136. doi: 10.5547/01956574.35.2.6

Faruqui, A., Sergici, S., and Akaba, L. (2014). The impact of dynamic pricing on residential and small commercial and industrial usage: New experimental evidence from Connecticut. The Energy Journal 35, 137–160. doi: 10.5547/01956574.35.1.8

Grimm, M., Lenz, L., Peters, J., and Sievert, M. (2020). Demand for off-grid solar electricity: Experimental evidence from Rwanda. J. Assoc. Environ. Resour. Econ. 7, 417–454. doi: 10.1086/707384

Harold, J., Lyons, S., and Cullinan, J. (2018). Heterogeneity and persistence in the effect of demand side management stimuli on residential gas consumption. Energy Econ. 73, 135–145. doi: 10.1016/j.eneco.2018.04.034

Wang,W., Ida, T., and Shimada, H. (2020). Default effect versus active decision: Evidence from a field experiment in Los Alamos. Eur. Econ. Rev. 128, 103498. doi: 10.1016/j.euroecorev.2020.103498

Wolak, F. A. (2011). Do residential customers respond to hourly prices? Evidence from a dynamic pricing experiment. Am. Econ. Rev. Papers Proc. 101, 83–87. doi: 10.1257/aer.101.3.83

Yoeli, E., Hoffman, M., Rand, D. G., and Nowak, M. A. (2013). Powering up with indirect reciprocity in a large-scale field experiment. Proc. Nat. Acad. Sci. U. S. A. 110, 10424–10429. doi: 10.1073/pnas.1301210110
